# Heart disease prediction with a feature-sensitized interpretable framework for the Internet of Medical Things sensors

**DOI:** 10.3389/fdgth.2025.1612915

**Published:** 2025-10-01

**Authors:** Nallakaruppan Kailasanathan, Gangadevi Ezhilarasan, Shitharth Selvarajan, Rajesh Kumar Dhanaraj, Dragan Pamucar, Nathan Shankar

**Affiliations:** ^1^ Balaji Institute of Modern Management, Sri Balaji University, Pune, India; ^2^ Department of Computer Science, Loyola College, Chennai, India; ^3^ School of Built Environment, Engineering and Computing, Leeds Beckett University, Leeds, United Kingdom; ^4^ Department of Computer Science and Engineering, Chennai Institute of Technology, Chennai, India; ^5^ Centre for Research Impact & Outcome, Chitkara University Institute of Engineering and Technology, Chitkara University, Rajpura, Punjab, India; ^6^ Symbiosis Institute of Computer Studies and Research (SICSR), Symbiosis International (Deemed University), Pune, India; ^7^ Széchenyi István University, Győr, Hungary; ^8^ Department of Electrical and Electronics Engineering, The University of Manchester, Manchester, United Kingdom

**Keywords:** XAI, LIME, SHAPELY, Random Forest, PDP, heart failure prediction, heart disease

## Abstract

**Introduction:**

Cardiovascular health is increasingly at risk due to modern lifestyle factors such as obesity, smoking, stress, hypertension, and sedentary behavior. Post-pandemic health practices and medication side effects have further contributed to rising cases of early heart failure, particularly among individuals aged 25–40 years. This highlights the need for an automated and interpretable framework to predict heart disease at an early stage.

**Methods:**

In this study, body vitals acquired from a secondary dataset. Machine learning models including Support Vector Machine, Random Forest, Decision Tree, and Logistic Regression were employed for classification. Model performance was evaluated using accuracy, F1-score, and k-fold cross-validation.

**Results:**

Among the tested models, the Random Forest classifier demonstrated superior performance with an accuracy and F1-score of 0.955. The interpretability is enhanced with model predictions were explained using Local Interpretable Model-Agnostic Explanations (LIME) for local surrogates and SHAP values for global surrogates. SHAP decision plots provided clear insights into classification behaviour and feature contributions.

**Discussion/Conclusion:**

The proposed interpretable machine learning framework successfully predicts heart disease with high accuracy while maintaining transparency in decision-making. With the integration of sensor data with cloud-based analysis and explainable AI techniques, this study contributes to reducing the incidence of early heart failures and supports more reliable decision-making in healthcare applications.

## 1 Introduction

Cardiovascular disease (CVD) has always posed a serious threat to human beings and remains the primary cause of death globally. Heart diseases can cause substantial risk to the life of a person and significantly impact human health and wellbeing (1). The World Health Organization (WHO) has reported that 18 million persons die of CVD every year, which represents 32% of all deaths worldwide, of which 85% are due to heart attacks. The WHO has stated that more than 70% of heart diseases occur in developing countries (2). The World Heart Federation has predicted nearly 23 million CVD-related deaths by 2030, and the American Heart Association has reported that by 2035, nearly 130 million adults will contract heart diseases (3). Heart diseases encompass various conditions such as irregular heartbeats, cardiomyopathy, arrhythmia, and peripheral or coronary artery that affect the heart and cause a global hazard health with serious medical manifestations (4, 5). The crucial risk factors for cardiovascular diseases are tobacco use, alcohol, unhealthy diet, physical inactivity, obesity, high blood pressure, diabetes, high cholesterol, and emotional implications (6). WHO has been deliberately making efforts to curtail the encumbrance of heart diseases by implementing prevention and control efforts. Members of the WHO are planning to facilitate drug and counseling treatments for a minimum of 50% of people with a high risk of cardiovascular disease by the end of this year (7). COVID-19 is an infectious pathogen that has created an aberrant impact on public health worldwide. It has been observed that COVID-19 infection has created an independent risk factor for heart diseases in some patients and has caused severe damage to the heart muscles, leading to myocarditis or heart failure. Blood clots and cardiac arrhythmias are the major risk factors for elevated mortality risks. Many emerging pieces of evidence and observational studies have been reported by researchers to the effect patients infected with the COVID-19 had suffered from impairment of myocardial function (8, 9) and cardiovascular complications (10, 11) such as myocarditis, arrhythmias, pericarditis, myocardial infarction, thromboembolism, stroke, and sudden death (12, 13). More than 72.3% of people, or over 5.55 billion individuals worldwide, have been administered a dose of COVID-19 vaccination to protect against virus variants effectively. However, vaccination intake rates have substantially stagnated for several reasons, and one among them is vaccine hesitancy (14, 15). The main reason for the reluctance on the part of people to get the vaccines administered is the side effects associated with cardiac complications like myocarditis and pericarditis (16). The vaccine side effects are associated with a high risk of myocarditis that is highest in males between the ages of 16 and 24 years (17). The studies conducted (18) show that males who received the second dose of the COVID-19 vaccine had the highest rate of cardiovascular complications (19). The Center for Disease Control and Prevention (CDC) is continuously monitoring and conducting various surveys on patients having symptoms such as chest pain, palpitation, pounding heart, and shortness of breath and advising them to take the related medical tests for diagnosing myocarditis and pericarditis (20). Monitoring patients with a high risk of heart disease is paramount to ensure their wellbeing and optimize treatment outcomes. Regular health monitoring helps clinicians to effectively assess the patient’s health condition, medical adherence, and drug intake adjustments. Health monitoring also helps patients understand their health conditions, disease progression, treatments, and self-care health management and prevents them from having the risk of adverse events (21).

The Internet of Medical Things (IoMT) is a transformative and revolutionary technological concept in the field of healthcare used for amalgamating medical resources connected with network technologies for monitoring, predicting, and preventing health-related diseases (22). The prognostic potential of the IoMT has fascinated the healthcare industry in terms of facilitating real-time surveillance using smart medical devices connected with software applications. The IoMT captures real-time data on patients using wearable devices, remote monitoring devices, connected medical equipment, implantable medical devices, mobile health applications, smart home medical devices, and point-of-care testing devices (23). The IoMT devices play a vital role in health monitoring, data collection, personalized care, and transmission of real-time data for the decision-making process by healthcare providers. The communication system in the IoMT enables connectivity and data exchange among patients and healthcare providers for improving the patient’s health condition and enhancing overall healthcare management (24). The IoMT is used in this work to monitor the patient’s health-related risk factors for cardiovascular diseases, such as tobacco use, use of alcohol, diet, physical activity, obesity, high blood pressure, diabetes, high cholesterol, and emotional implications for diagnosing, monitoring, and preventing heart diseases. Smart medical devices track the heart rate, electrocardiogram (ECG), heart rhythms, heart’s electrical activity, blood pressure, insulin level, sleeping level, physical activity, and stress management (22).

The integration of IoMT technology into Artificial Intelligence has the potential to optimize the healthcare decision-making process by analyzing real-time data to develop predictive models (25–28). The emerging utilization of AI and machine learning (ML) models has the potential to revolutionize healthcare management by enabling automation and analyzing data from IoMT devices for identifying symptoms and improving decision outcomes (29). AI models predict disease progression, detect abnormalities and risk patterns, and facilitate interventions to avert adverse events (30). However, AI approaches are often called “black boxes” due to a lack of interpretability and accountability. The high dimensionality feature of AI techniques makes it tedious for humans to interpret and understand the decisions taken (31). Patients can face difficulty understanding the decisions and insights the machine learning models produce. Explainable AI (XAI) is considered a magic box to counteract the black box nature of AI models by providing optimal solutions with transparency in the healthcare industry (32). The cutting-edge XAI technology is a game changer by as it generates explanations, visualizations, and justifications for the decision outcomes produced by AI models (33). The healthcare industry can utilize this trailblazing XAI model to make clinical decisions transparently by allowing healthcare providers and patients to interpret the underlying reasoning behind AI’s decisions. An XAI model can be used for treatment recommendation plans that help physicians understand and comprehend appropriate interventions. This improves the trustworthiness among healthcare providers and patients for the successful implementation and deployment of AI-based healthcare systems (34).

The purpose of this study is to develop an accurate and interpretable XAI framework to predict heart disease using input received through the IoMT sensors. This study also clearly understands the importance of medical parameters that provide transparency and consistency in the predictions. In this study, Section 2 describes the review of the literature on the prediction of heart disease on existing works. Section 3 discusses the materials and methods where a description of datasets, system architecture, and a mathematical model is added. Section 4 presents the results and Section 5 provides a discussion of the results. Finally, the paper concludes with a comprehensive conclusion section.

## 2 Background

**Figure 1:**
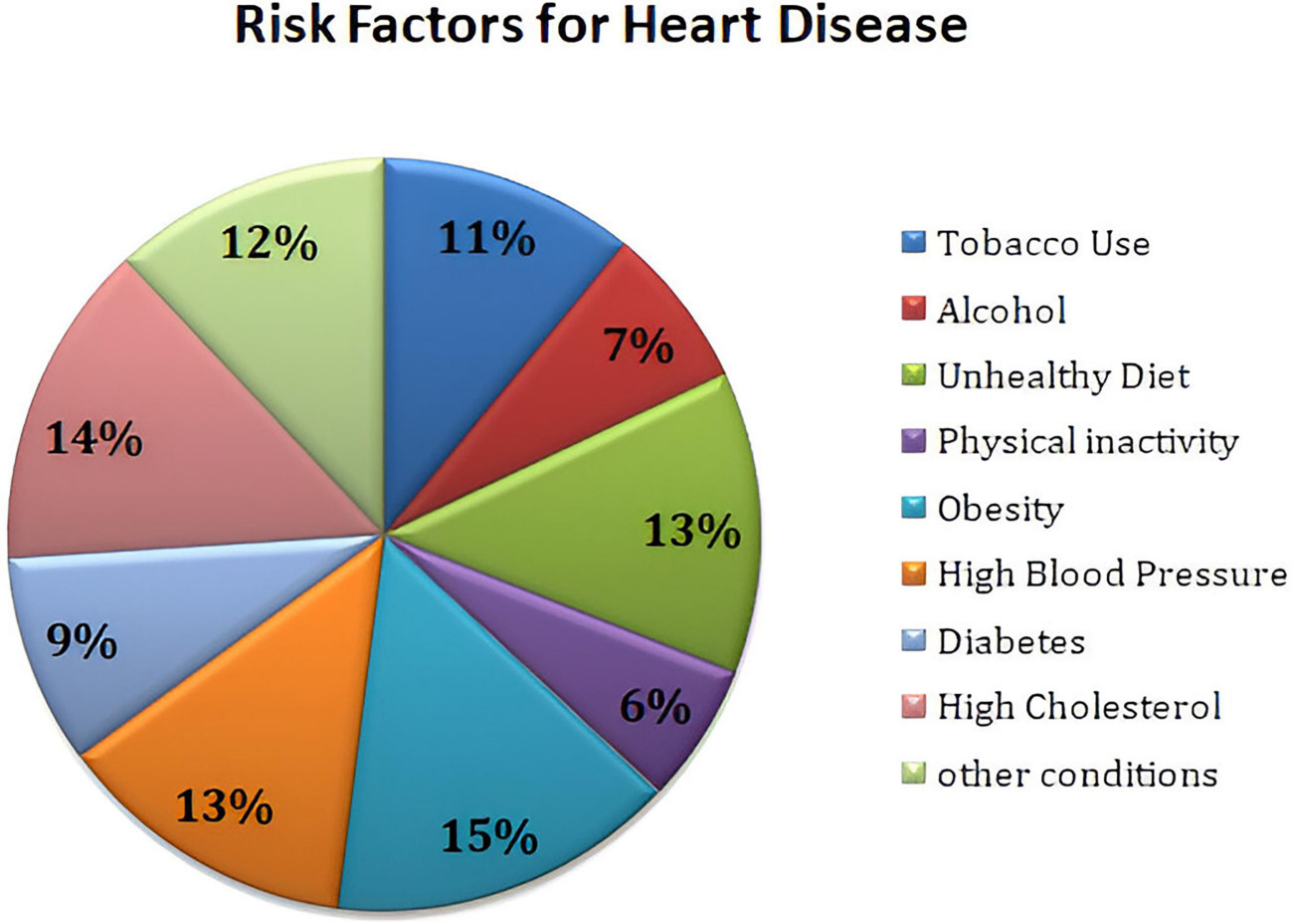
Heart disease risk factors.

**Figure 2:**
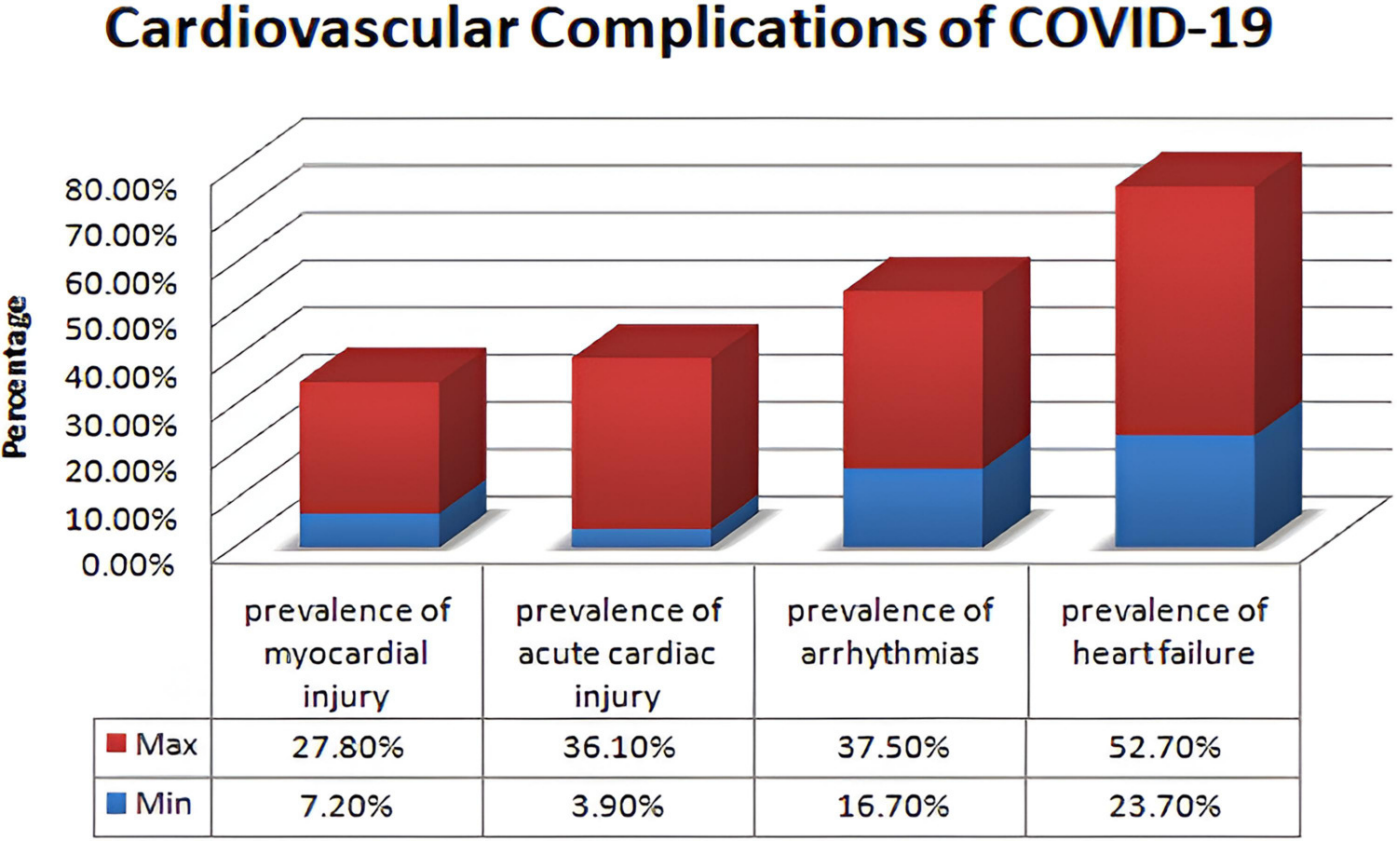
Cardiovascular complications of COVID-19.

The foundation for the enrichment of research is based on a comprehensive literature survey and relevant investigation in the respective domain. This section focuses on several research methodologies and literature reviews on heart disease prediction, IoMT-based health monitoring, machine learning models, and XAI. The current research methodologies and their respective strengths are identified along with their limitations. Heart diseases are considered a potential threat to human life and a leading reason for morbidity and mortality. The WHO has outlined the critical risk factors of heart diseases such as tobacco use, alcohol, unhealthy diet, physical inactivity, obesity, high blood pressure, diabetes, high cholesterol, and emotional implications depicted in Figure 1. A meta-analysis reveals various cardiovascular complications associated with COVID-19 and vaccinations. Figure 2 illustrates the cardiovascular disease complications of COVID-19 and notes the prevalence of myocardial injury, acute cardiac injury, arrhythmias, and heart failure, all of which elevates the risk of mortality.

### 2.1 Related works

Kumar et al. (35) analyzed the classification of heart diseases prediction, which involved five stages such as heart disease detection and diagnostics, machine learning models and algorithms used for healthcare, feature engineering and optimization techniques, evolving and advanced techniques in healthcare, and different applications of AI across various diseases and health conditions. This study analyzed the deep learning models for early diagnosis of heart disease prediction with evaluation techniques like sensitivity, specificity, and area under the curve (AUC). It also discusses ethical issues, dataset challenges, and transparency of the model. This paper clearly pointed out the advantages and challenges of modern equipment and advanced prediction systems. Rajkumar et al. (36) proposed IoT-based framework and advanced and enhanced deep learning framework. A Hungarian heart disease dataset is used, which is preprocessed by a median studentized residual approach for reducing error values and missing data. The Harris Hawk Optimization (HHO) approach is applied to select the features during preprocessing, which are classified using Modified Deep Long Short-Term Memory (MDLSTM). This output is updated by the Improved Spotted Hyena Optimization (ISHO) algorithm and achieved 98.01% accuracy in implementation. Hammadi et al. (37) presented an updated framework for cardiovascular disease prediction with the hybrid ensemble learning method. A soft voting method is introduced. Class imbalance is given preference here and the model achieves 97.4% accuracy in score 1, 83.6% accuracy in score 2, and 93% accuracy in score 3. This advanced technique applies ensemble learning for early detection of heart diseases. The Department of Computer Science & Engineering BRAC University in Dhaka, Bangladesh. Rokoni et al. (38) focused on model interpretability and applied one-dimensional Convolutional Neural Networks (1D CNNs) and logistic regression for classifying diseases. It achieves 80% overall accuracy, and Local Interpretable Model-Agnostic Explanations (LIME) provides transparency by finding the influenced features like glucose, blood pressure, and troponin.

Wang and Song (39) presented an edge-assisted IoMT framework for monitoring aged people having chronic diseases. The IoMT-based smart home monitoring model is utilized to access medical data and diagnose diseases for aged persons by continuously monitoring and communicating hastily using edge computing. Martinek et al. (40) used federated learning and blockchain technology to ensure privacy for healthcare monitoring. The IoMT technology used in this work employs sensors for health monitoring, and the sensed data are stored and managed using a fog-cloud-assisted network. The federated learning and fraud detection mechanism–enabled blockchain framework is used to process application workloads and validate the quality of service. Kumar et al. (41) proposed a novel IoMT-based healthcare monitoring system using rooted elliptic curve cryptography with Vigenere cipher (RECC-VC) for securing the environment. RECC-VCC enhances security, and the exponential K anonymity model is used for privacy protection. The Improved Extension Neural Network (IENN) framework is used to analyze the level of sensitive data, and the Gaussian mutated chimp optimizer is used to update the weight. Blockchain technology is used to store and manage transactions on the cloud server. Kishor and Chakraborty (42) presented an IoT-based health monitoring system by predicting various diseases such as heart disease, diabetes, breast cancer, dermatology, thyroid, liver disease, and surgery data using machine learning approaches like Decision Tree, Naïve Bayes, Random Forest, Support Vector Machine (SVM), Adaptive Boosting, Artificial Neural Network, and K-nearest neighborhood (KNN). Shafiq et al. (43) presented a deep learning framework using CNN for detecting heart disease symptoms by analyzing the biosensor input for detecting heart disorders. The PASCAL dataset is used to train the CNN model, and the real-time data sensed by IoT sensors are stored in the cloud. The sound of the heart is given as input for classifying whether patients are affected by heart disease. Kumar and Gandhi (44) introduced a three-tier IoT architecture for detecting heart disease. Wearable sensors are used to observe the patient’s health condition, and Apache HBase stores the patient’s monitored data in cloud computing. Apache Mahout is utilized to implement a logistic regression framework for heart diseases. Panja et al. (45) utilized IoMT architecture to monitor and assess the health issues of infected patients and trigger an alert message to their clinicians and relatives. The real-time data collected from patients are transmitted to the cloud via edge devices for processing. A severity analysis of the infection is carried out using fuzzy logic to detect the risk status of COVID-19 patients effectively. Jain et al. (46) proposed point-of-care testing to rapidly detect infectious diseases and give spot results for taking early action.

IoMT devices are also used to capture the patient’s vitals for early detection of diseases such as malaria, influenza A, Ebola virus, Zika virus, COVID-19 virus, and dengue fever. Rezaee et al. (47) suggested a meta-heuristic fuzzy inference system for emotion recognition using the IoMT. Patients are tested by playing music videos to detect their emotional states. Electroencephalography (EEG) signals are captured before and after meditation. Using an optimized, innovative Gunner algorithm, a fuzzy inference-based classification approach is used to classify emotions. Krupa et al. (48) presented an IoMT-based deep learning framework for automatically detecting fetal QRS. The framework used two methods: one for detecting fetal QRS complex using a deep neural network and the second for classifying the results by acclimatizing transfer learning to improve accuracy. The method uses a time-frequency image as input for an IoT-based deep neural network in the abdominal ECG without removing the maternal components. Lu et al. (49) developed a novel IoMT-based fetal monitoring model incorporating an automatic fetal heart rate (FHR) rating method to evaluate fetal health conditions inside the uterus using digital cardiotocographic signals. The monitoring system uses Kreb’s Fischer, improved Fischer, and American College of Obstetricians and Gynecologists (ACOG) classifiers to detect and classify fetal conditions as good and bad for comparison. Rahmani et al. (50) used fog computing–based e-Health gateways by offering higher-level services for storage provisioning and data processing to form a geo-distributed middle layer between the cloud and IoT sensors. The framework uses an early warning score for monitoring health to facilitate energy efficiency, interoperability, reliability, mobility, and performance. Nandy et al. (51) proposed an IoMT-based intelligent agent mechanism to detect brain response using an electroencephalography signal. A bag of neural network categorizes the complex brain signals captured by the IoMT sensors and detects the brain responses. The IBoNN framework is compared with standard machine learning algorithms. Yadav et al. (52) presented biomarker-based electrochemical immuno sensors for diagnosing COVID-19 using the IoMT and artificial intelligence. The smart sensing technique is used with bioinformatics approaches for monitoring non-invasive SARS-COV2.

**Figure 3:**
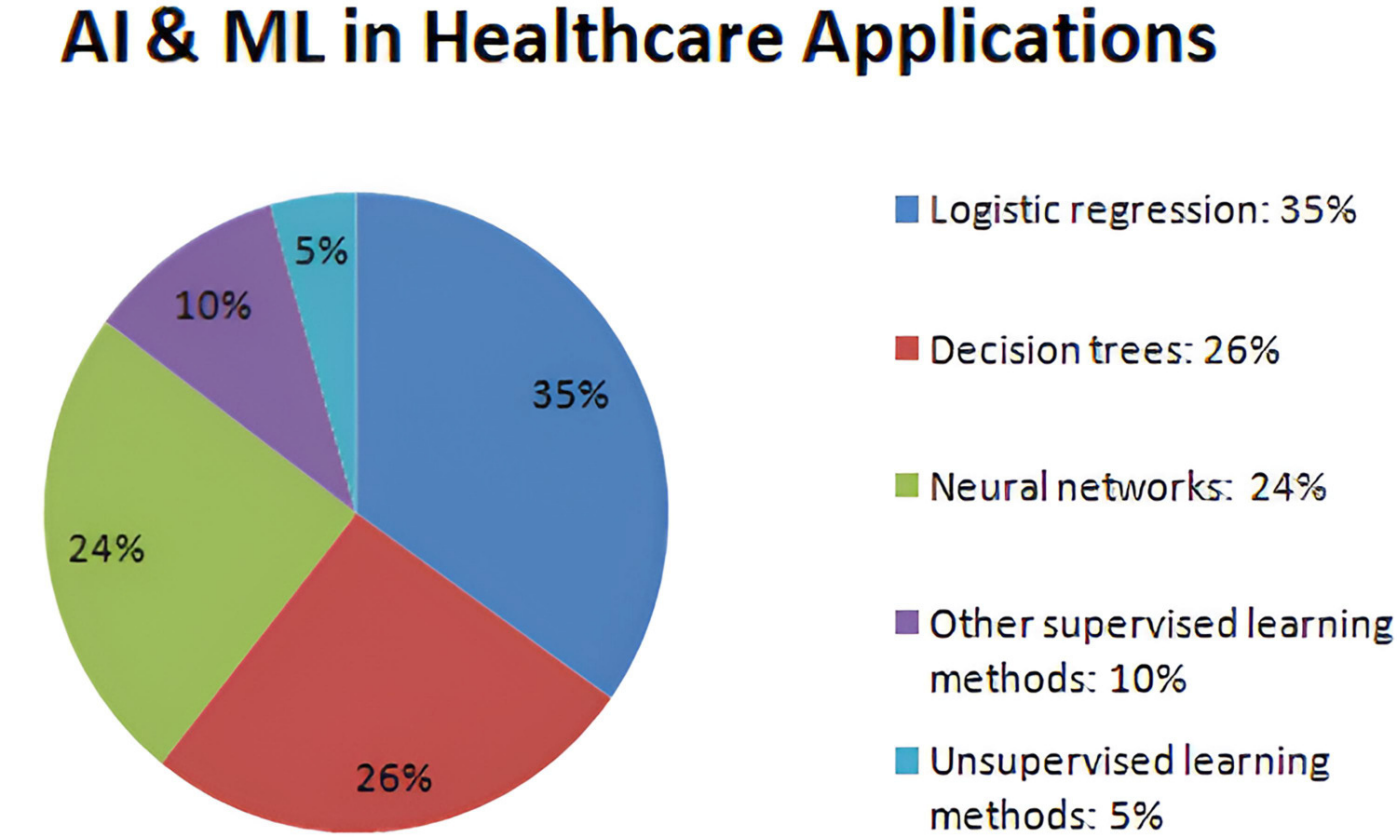
AI and machine learning techniques used for healthcare applications.

Verma et al. (53) summarized nano-integrated wearable biosensors and the use of 5G in the Internet of Things for healthcare applications. Fouad et al. (54) presented a numerical approach using the Gautschi model for vertebral tumor prediction. The IoMT technology is used for predicting tumors employing heuristic hock transformation for evaluating possible perpetual incapacity caused by tumors on Haar-like characteristics (HLC), logistics models (LM), conservative therapy method (CTM), and carbon fiber including reinforced materials (CFRM) approaches. Figure 3 describes the percentage of AI and machine learning approaches used in the healthcare industry. In recent years, machine learning models have revolutionized the diagnosis of various diseases and the assessment of the risk factors involved in making accurate decisions. According to a background study, supervised learning approaches like 35% of logistic regression, 26% of decision tree, and 24% of neural networks, as well as 5% of unsupervised learning methods like clustering and anomaly detection, have been used by the healthcare industry to assess the risks.

Ribeiro et al. (55) presented a novel agnostic approach called LIME, which is used to provision the comprehensiveness of the decisions made by the banking staff by determining them with simple and interpretable models. The LIME model helps improve accuracy, transparency, and trustworthiness. This model can be used with complex machine learning algorithms without any knowledge of their working mechanisms. Lundberg and Lee (56) proposed a new technique, SHAPELY Additive exPlanations (SHAP), to interpret the existing complex machine learning models. The SHAP algorithm provides global and local interpretations to help borrowers easily understand the predictions made by black box techniques. SHAP can be used in various machine learning approaches and deep neural networks and effectively work with real-world datasets.

Muddamsetty et al. (57) presented an evaluation for understanding the outcomes of machine learning models. Thus, it is evident that the XAI model helps to present outcomes with transparency and provides solutions to black box models. Explanations for the clinical prediction outcome entail the justification of reliability and trustworthiness that can be achieved using XAI models (58). Onan (59) presented a hierarchal graph-based model for text classification of dynamic fusion using BERT. The framework uses seven stages for graph-based text classification and analysis with various benchmark functions. Onan (60) proposed a genetic technique combined with graph-based neural networks for generating augmented text having high dimensional feature space. The objective function is based on perplexity when evaluating the quality of generating augmented text data. Onan (61) proposed a Semantic Role Labeling algorithm with an Ant colony optimization approach for generating training data to improve the performance of the natural language processing (NLP) framework. The semantic roles are identified using semantic role labeling (SRL) for text augmentation to enhance the quality of training data. Onan (62) suggested a bidirectional convolutional recurrent neural network framework for semantic analysis using gated recurrent unit (GRU) and LSTM layers. Feature extraction is carried out by the bidirectional layers to reduce dimensionality and extract high-quality features. Onan et al. (63) presented a two-stage topic extract model using a word embedding approach and cluster analysis. The word vectors are extracted by Word2Vec, POS2vec, LDA2vec, and word position2vec schemes. A comparison of Naïve Bayes, SVM, Random Forest, and Logistic regression with ensemble methods is used for evaluating the statistical key extraction model (64). Onan et al. (65) presented a consensus cluster mechanism using an undersampling model with five supervised learning algorithms and three ensemble learners for imbalanced learning. Onan and Korukoğlu (66) utilized an ensemble model for feature selection with a genetic-optimized algorithm for sentimental analysis. Sentiment classification based on a hybrid ensemble pruning model with consensus clustering is utilized for text classification (67). Sentimental analysis for product reviews (68), online course evaluation (69), and mining opinions for instructors (70) is done using deep neural networks. Onan (71) presented a comparative analysis of feature engineering models using five base learners for text genre classification and language function analysis. Onan and Toçoğlu (72) suggested inverse gravity moment utilizing bidirectional LSTM for representing text documents. The LSTM framework is evaluated on the basis of the sarcasm identification corpus. The deep learning model is utilized to identify sarcasm for predicting the performance of sentiment analysis. Vakharia et al. (73) proposed three deep learning frameworks with optimized explainable artificial intelligence for predicting the discharge capacity of the battery. The jellyfish optimization algorithm is used with the XAI model to improve the predictive performance. Ali et al. (74) presented an SVM model based on four Ant Bee Colony (ABC) algorithms, Genetic Algorithm (GA), Particle Swarm Optimization (PSO), and Whale Optimization Algorithm (WAO) for tuning hyperparameters. A teaching learning–based optimization algorithm with a heat transfer searching model is used to select features and identify faults. Suthar et al. (75) and Vakharia et al. (76) highlighted a comparative study of feature ranking approaches for fault identification by using the Fisher score, ReliefF, Gain ratio, Wilcoxon rank, and Memetic feature selection model. The literature survey shows that the XAI framework improves prediction accuracy with interpretability and explainability in healthcare applications because of the crucial nature of the decision-making process and public health safety. Tables 1, 2 depict a comparison of various black box models used for healthcare applications.

**Table 1:** Motivation for the proposed work from the review perspective.

Reference	Title	Advantages	Research gap
Hashem et al. (77)	Predicting neurological disorders linked to oral cavity manifestations using IoMT-based optimized neural networks	• Reduced complexity • Oral cavity linked nervous problem detection rate • Minimized the feature dimension	• Interpretability vs. accuracy trade-off • Limited explanation of complex models • Scalability
Zhu et al. (78)	IoMT-enabled real-time blood glucose prediction with deep learning and edge computing	• The wearable sensor’s power and memory footprint are analyzed • Prediction accuracy for three datasets • Scalability.	• Limited applicability • Interpretability vs. accuracy trade-off
Abbas et al. (79)	Secure IoMT for disease prediction empowered with transfer learning in healthcare 5.0, the concept and case study	• Model performance • Generalizability • Security	• Limited explanation of complex models • Scalability
Nandy et al. (80)	An intrusion detection mechanism for a secure IoMT framework based on swarm-neural network	• Security. High performance due to optimization	• Limited applicability • Interpretability vs. accuracy trade-off • Scalability
Lakhan et al. (81)	Federated learning–based privacy preservation and a fraud-enabled blockchain IoMT system for healthcare	• Privacy preservation • Minimum energy consumption	• Limited number of models • Lack of external validation • Limited scope of interpretability
Wang and Song (39)	An edge-assisted IoMT-based smart-home monitoring system for the elderly with chronic diseases	• Local medical data diagnosis and rapid communication • Scalability	• Limited applicability • Interpretability vs. accuracy trade-off
Zhang et al. (82)	A joint deep learning and internet of medical things–driven framework for elderly patients	• Energy efficiency • Sustainability • Reliability during data transmission	• Limited to a specific set of datasets • Does not incorporate interpretability techniques • Does not incorporate explainability techniques
Khan and Algarni (83)	A healthcare monitoring system for the diagnosis of heart disease in the IoMT cloud environment using MSSO-ANFIS	• Better accuracy • Improved convergence rate	• Lack of external validation • Limited scope of interpretability
Guleria et al. (84)	XAI framework for cardiovascular disease prediction using classification techniques	• Comprehensive evaluation, • Large dataset • Transparent evaluation criteria	• Lack of external validation • Limited scope of interpretability • Limited to a specific set of datasets

**Table 2:** Comparison of algorithms and prediction performance.

Reference	Algorithms compared	Type of data	Prediction performance
Juhola et al. (85)	ANN, NB	Disease symptom	Accuracy: (ANN=85, NB=88)
Long et al. (86)	ANN, LR	Clinical and demographic data	Accuracy: (ANN=0.965, LR=0.963)
Palaniappan and Awang (87)	ANN, DT, SVM	Clinical data for cancer incidence and survival	Accuracy: (ANN=0.947, DT=0.936, SVM=0.957)
Jin et al. (88)	LR, RF	Electronic health records	Accuracy: (LR=0.663, RF=0.627)
Puyalnithi and Viswanatham (89)	DT, NB, RF, SVM	Clinical and demographic data	Sensitivity: (ANN=0.956, DT=0.958, SVM=0.971)
Forssen et al. (90)	LR, RF	Metabolomic data	Accuracy: (LR=0.767, RF=0.732)
Tang et al. (91)	ANN, LR	Clinical, demographic, behavioral, and medical data	Specificity: (ANN=0.928, DT=0.907, SVM=0.945)
Toshniwal et al. (92)	ANN, LR	Clinical and demographic data	Accuracy: (ANN=0.909, LR=0.897)
Yang et al. (93)	ANN, DT, LR	Clinical and demographic data	Accuracy: (ANN=0.909, DT=0.935, LR=0.894)
Mustaqeem et al. (94)	DT, RF, SVM	Image data	Accuracy: (DT=0.932, RF=0.963, SVM=0.959)
Mansoor et al. (95)	DT, KNN, NB	Electronic health records, medical image, and gene data	Accuracy: (DT=0.646, KNN=0.454, NB=0.495)
Kim et al. (96)	LR, NB, SVM	Gut microbiota	Accuracy: (LR=0.98, NB=0.94, SVM=0.99)
Taslimitehrani et al. (97)	ANN, LR, SVM	Electrochemical measurements of saliva	Accuracy: (ANN=80.70, LR=75.86, SVM=84.09)
Anbarasi et al. (98)	DT, NB	Clinical and demographic data	Accuracy: (DT=99.2%, NB=96.5%)
Bhatla and Jyoti (99)	ANN, DT, NB	Clinical data	F1-score: (ANN=80.20, LR=75.71, SVM=84.06)
Thenmozhi and Deepika (100)	KNN, LR, SVM	Demographic, anthropometric, vital signs, diagnostic, and clinical laboratory measurement data	Accuracy: (KNN=79.5, LR=80.7, SVM=82.6)
Tamilarasi and Porkodi (101)	KNN, LR, NB, RF, SVM	Demographic and clinical test result	Accuracy: (KNN=0.721, LR=0.755, NB=0.762, RF=0.803, SVM=0.749)
Marikani and Shyamala (102)	ANN, LR, RF, SVM	Demographic, anthropometric, diagnostic and clinical lab measurement data	Accuracy: (ANN=0.931, LR=0.935, RF=0.930, SVM=0.986)
Lu et al. (103)	ANN, NB, SVM	Clinical, demographic, and diagnostic data	Accuracy: (ANN=86.04, NB=82.31, SVM=86.62)

ANN, artificial neural network; NB, Naïve Bayes; LR, logistic regression; DT, decision tree; RF, random forest.

### 2.2 Research questions

This study aims to answer all the following questions:

1. How can this IoMT sensor device help predict the heart disease effectively?
2. Which type of machine learning or deep learning methodologies are most relevant for predicting heart disease accurately?
3. How can feature selection techniques help identify the most dominant parameters for prediction?
4. What are all the challenges in integrating the IoMT with real-time heart disease prediction and how can they be addressed?
5. Does the proposed framework apply effectively in real-time analysis problems for early heart disease prediction?

This work addresses the drawbacks of state-of-the-art ML-based techniques in achieving increased transparency, interpretability, and accountability with high-accuracy outcomes.

### 2.3 Feature selection and existing work

**Figure 4:**
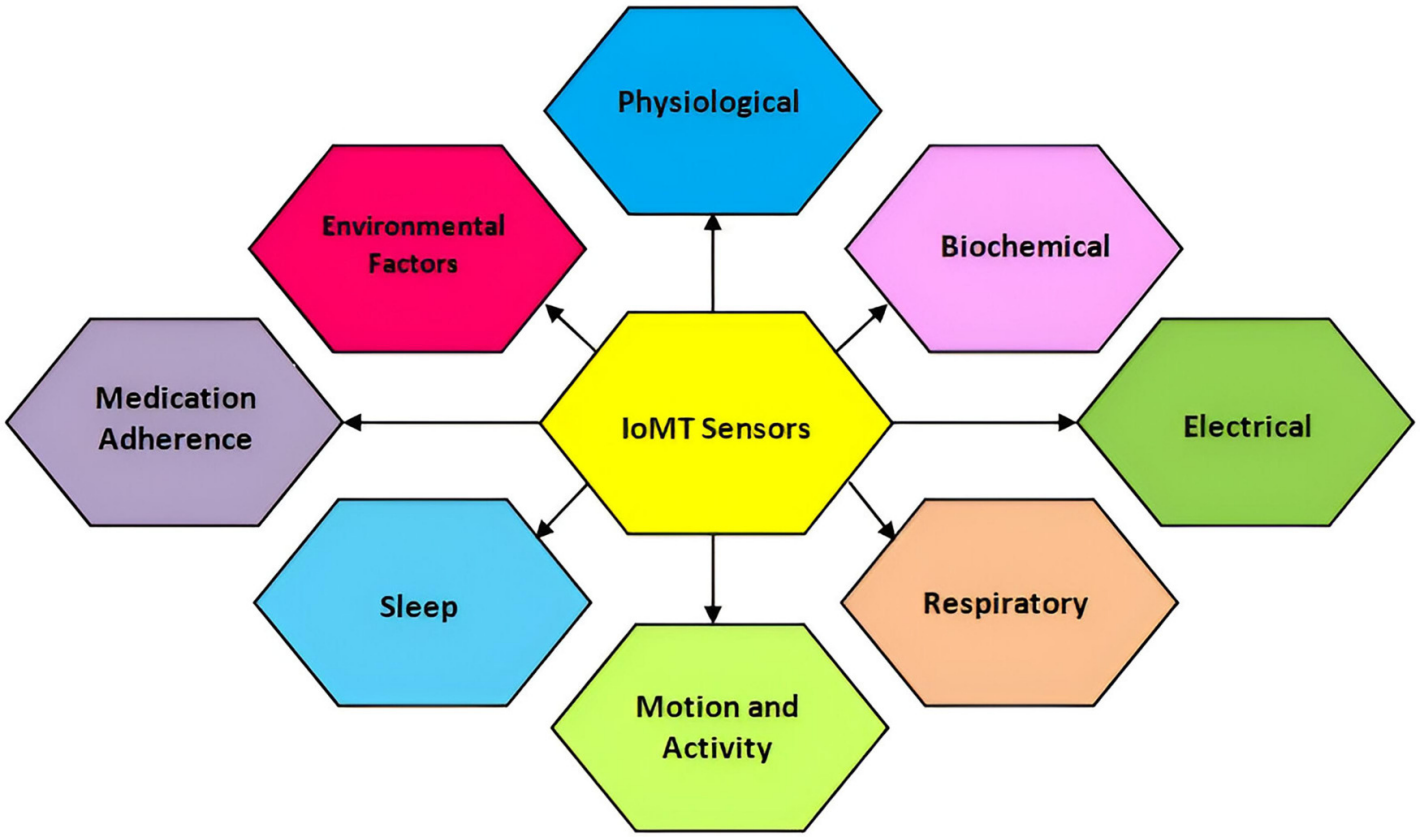
Target features of IoMT sensors.

The IoMT integrates wearable devices with sensor technologies to monitor health parameters, track physical activity, and enable remote patient monitoring. The literature survey focuses on two specific sensor categories: vital signs and motion sensors. For vital signs sensors, Rao et al. (104) present a non-invasive wearable device that accurately monitors BP without requiring invasive catheterization. The device utilizes capacitive wrist and/or foot sensors to acquire pulse waveform data, which are then processed using artificial neural networks to determine systolic, diastolic, and mean arterial pressures. A comparison with invasive arterial line data confirmed the device’s accuracy, making it a viable alternative for continuous BP monitoring in critically ill infants. In motion sensors, Jakob et al. (105) evaluate the effectiveness of wearable sensors in analyzing motion patterns in individuals with Parkinson’s disease. The study assesses the accuracy and reliability of the sensor system in detecting and quantifying motor symptoms associated with Parkinson’s disease, such as bradykinesia and shuffling gait. Wearable sensors distinguish Parkinson’s patients from healthy controls, showing their potential for clinically relevant gait assessments in flexible environments. These research papers are examples of studies conducted on sensors used in IoMT projects. The surveyed literature demonstrates the significance of vital signs sensors in non-invasive blood pressure monitoring and the potential of motion sensors in analyzing motor symptoms in Parkinson’s disease. The following parameters mentioned in Figure 4 are taken into consideration while designing existing and ongoing IoMT systems that are positively helping to transform the IoMT domain through cutting-edge technology. Physiological parameters, biochemical parameters, electrical activity, respiratory parameters, motion and activity, sleep patterns, environmental factors, and medical adherence are the IoT devices applied for treatments. Some of the IoMT projects have incorporated the above-mentioned parameters and have made the availability of diagnostics more accessible, for example, Ovularing (106), VitalPatch (107), SmartPill (108), GlucoWear (109), MindMotion Pro (110), BioStampRC (111), Biotricity (112), SmartMat (113), PillCam (114), WAND (Wireless Artifact-free Neuromodulation Device) (115), Abilify MyCite (116), Embrace (117), and Insulet Omnipod (118).

## 3 Materials and methods

**Figure 5:**
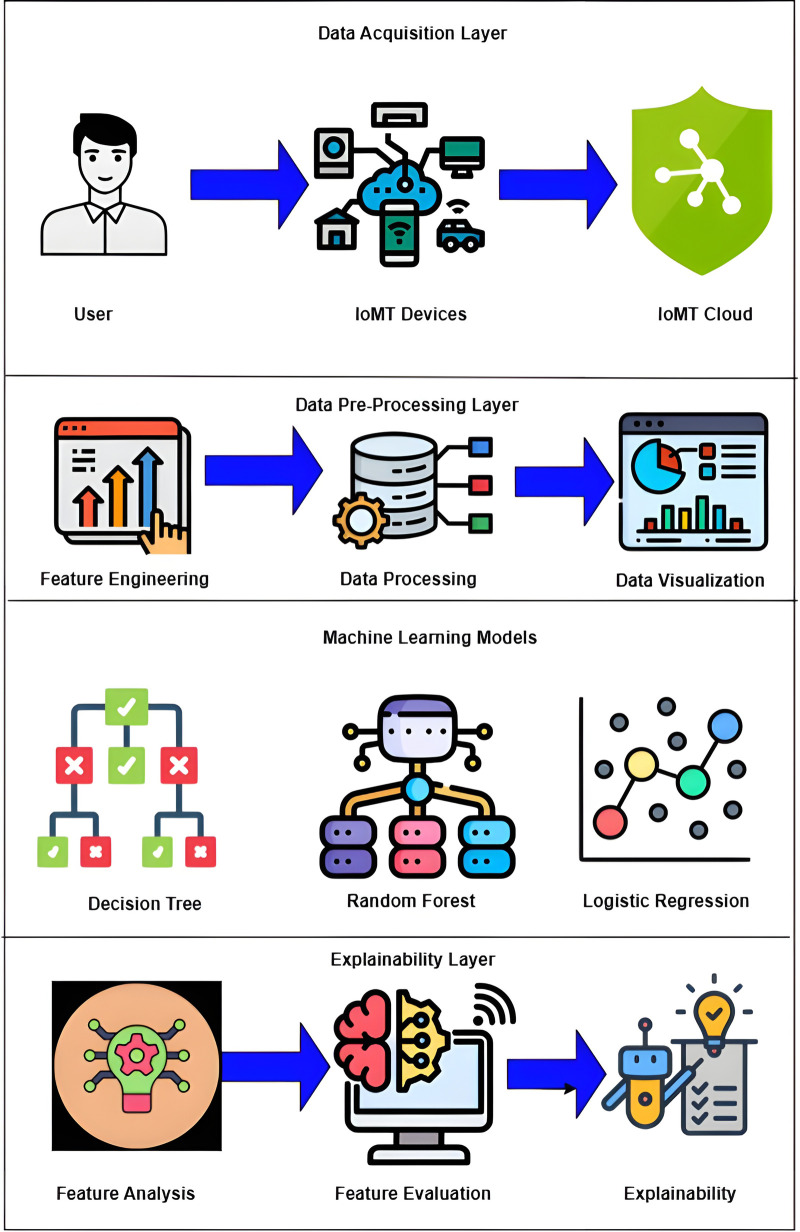
Layered architecture diagram of proposed work. Created using Draw.io.

In this work, we propose an IoMT-based heart disease prediction framework based on machine learning models like Logistic Regression (119), SVM (120), Decision Tree (121), Gradient Boost (122), and Random Forest (123). Figure 5 depicts the layered architecture of the work having four layers of IoMT: a device layer, cloud layer, machine learning models layer, and Explainable AI layer. The IoMT devices capture patients’ vitals, and the sensed patient’s data are transferred to cloud storage. The machine learning models herewith are used to detect and classify cardiovascular diseases and associated risk factors for diagnosing, monitoring, and preventing heart diseases. XAI techniques such as LIME (124) and SHAP (125) help overcome the limitations of traditional Machine Learning models by providing interpretable decision outcomes, thereby assisting both patients and clinicians.

### 3.1 Importance of XAI in the IoMT

The following case studies outline why XAI will prove to be a revolutionary change required in the IoMT.

#### 3.1.1 Case study 1: a 26-year-old adult died due to cardiac arrest

A 26-year-old man collapsed suddenly at a Metro Station in New Delhi because of cardiac arrest. The young man was immediately taken to a hospital, and the physician declared that the person died because of chronic fat deposits in the arteries. The *postmortem* was carried out at a medical institute, which revealed that the visceral organs and the brain were congested, resulting in lung blockage. Following this incident, the healthcare administration raised concerns regarding the prevalence of abrupt cardiac deaths among young adults and drew attention to the presence of undiagnosed cardiovascular diseases. Clinicians are advised to consider the risk factors and causes of heart disease and to take preventive measures for early diagnosis and further treatments.

#### 3.1.2 Case study 2: a 40-year-old actor’s demise due to massive cardiac arrest

A 40-year-old man and actor died because of a massive heart attack at his Mumbai residence. He took medicine, slept, and did not wake up. He was immediately taken to the Cooper Hospital in Mumbai, and the clinicians declared that the person was brought dead due to a massive heart attack. Clinicians worldwide are advised to assess the risk factors and lifestyle changes, thereby stressing regular health checkups that can help prevent cardiovascular diseases.

Table 3 provides an overview of the sensors described above and elucidates the shortcomings and advantages of these devices, which acted as a support to this work.

**Table 3:** Comparison of wearable and ingestible health devices.

Sl. no.	Type	Application	Parameters	Advantages	Merits	Demerits
1	Ovularing (106)	Wearable	Women’s health	Ovulation monitoring	Accurate fertility tracking	Limited compatibility with other devices
2	VitalPatch (107)	Wearable	Healthcare	Vital signs monitoring	Real-time health monitoring	Requires regular battery replacement
3	SmartPill (108)	Ingestible	Healthcare	Drug delivery monitoring	Non-invasive medication tracking	Possibility of device malfunction
4	GlucoWear (109)	Wearable	Diabetes care	Continuous glucose monitoring	Improved glucose management	Calibration requirements for accuracy
5	Bio Stamp RC (110)	Wearable	Research	Motion analysis	Long-term data collection	Limited sensor placement options
6	Biotricity (111)	Wearable	Cardiology	ECG monitoring	Real-time cardiac monitoring	Relatively high cost for consumer use
7	Mind motion pro (112)	Bio-feedback devices	Various rehabilitation applications	Muscle activity, EMG	Provides real-time feedback for muscle control	Relies on accurate sensor placement and signal quality
8	SmartMat (113)	Wearable	Fitness	Yoga and exercise tracking	Precise posture and movement analysis	Limited battery life
9	PillCam (114)	Ingestible	Medical imaging	Gastrointestinal imaging	Non-invasive imaging of the digestive system	Limited imaging capabilities compared with MRI
10	WAND (115)	Implantable	Neurology	Deep brain stimulation	Effective treatment for neurological disorders	Invasive surgical procedure for implantation
11	Abilify MyCite (116)	Ingestible	Mental health	Medication adherence	Monitors medication ingestion	Limited availability and regulatory approval
12	Empatica Embrace (117)	Wearable	Epilepsy	Seizure detection	Alerts caregivers during seizures	Some false alarms and limitations in accuracy
13	Insulet Omnipod (118)	Wearable	Diabetes care	Insulin delivery	Tubeless insulin pump system	Initial setup and learning curve for users

### 3.2 Dataset description

CVD takes the lives of around 18 million people every year and is the primary cause of death. The rate of accountability of death reports due to CVD is around 31%. A total of 80% of deaths associated with CVD are mainly due to heart attack and stroke. These attacks are observed in groups of people who are less than 70 years old. With this in mind, a dataset (126) has been prepared as an amalgamation of observations recorded from Cleveland (303), Hungarian (294), Switzerland (123), Long Beach, VA (200), and Stalog Dataset (270). After removing duplicates, the final dataset contains 918 instances with 11 important features for analyzing CVD diseases. The dependent target class is Heart Failure. The other independent features are Age, Sex, Chest Pain Type, ST_Slope, Cholesterol, Resting BP, Blood Sugar, Resting ECG, Exercise Angina, Old Peak, and Maximum Heart Rate (MaxHR). Some of the features are numeric, and some of the features are non-numeric. Table 4 provides the list of features converted to numeric data. The string data are transformed using the Label Encoder preprocessing technique with Min-Max scalar transformation. The dataset (126) has no missing values or class imbalance. Heart Rate, Variable Heart Rate, Blood Glucose Level, MaxHR, Blood Pressure, and ECG (Polar H10 Sensor) are measured by IoMT sensors. The other readings are observed in the oscilloscopes and treadmills (ST_Slope, Restring Angina), and some data are collected directly from patients and their attenders (Name, Age, Sex, etc.).

**Table 4:** Feature conversation details of the dataset.

Sl. no	Features	Types	Numeric change	Transformation
1	Chest pain type	ATA	1	Label encoder and min-max scalar
		NYP	2	
		ASA	3	
		TA	4	
2	ST_Slope	Up	1	Label encoder and min-max scalar
		Down	0	
		Down zero	−1	
3	Resting ECG	Normal	0	Label encoder and min-max scalar
		Abnormal	1	
4	Sex	Male	1	Label encoder and min-max scalar
		Female	0	
5	Exercise angina	Yes	1	Label encoder and min-max scalar
		No	0	

NYP, non-anginal pain; TA, typical angina.

### 3.3 System architecture

**Figure 6:**
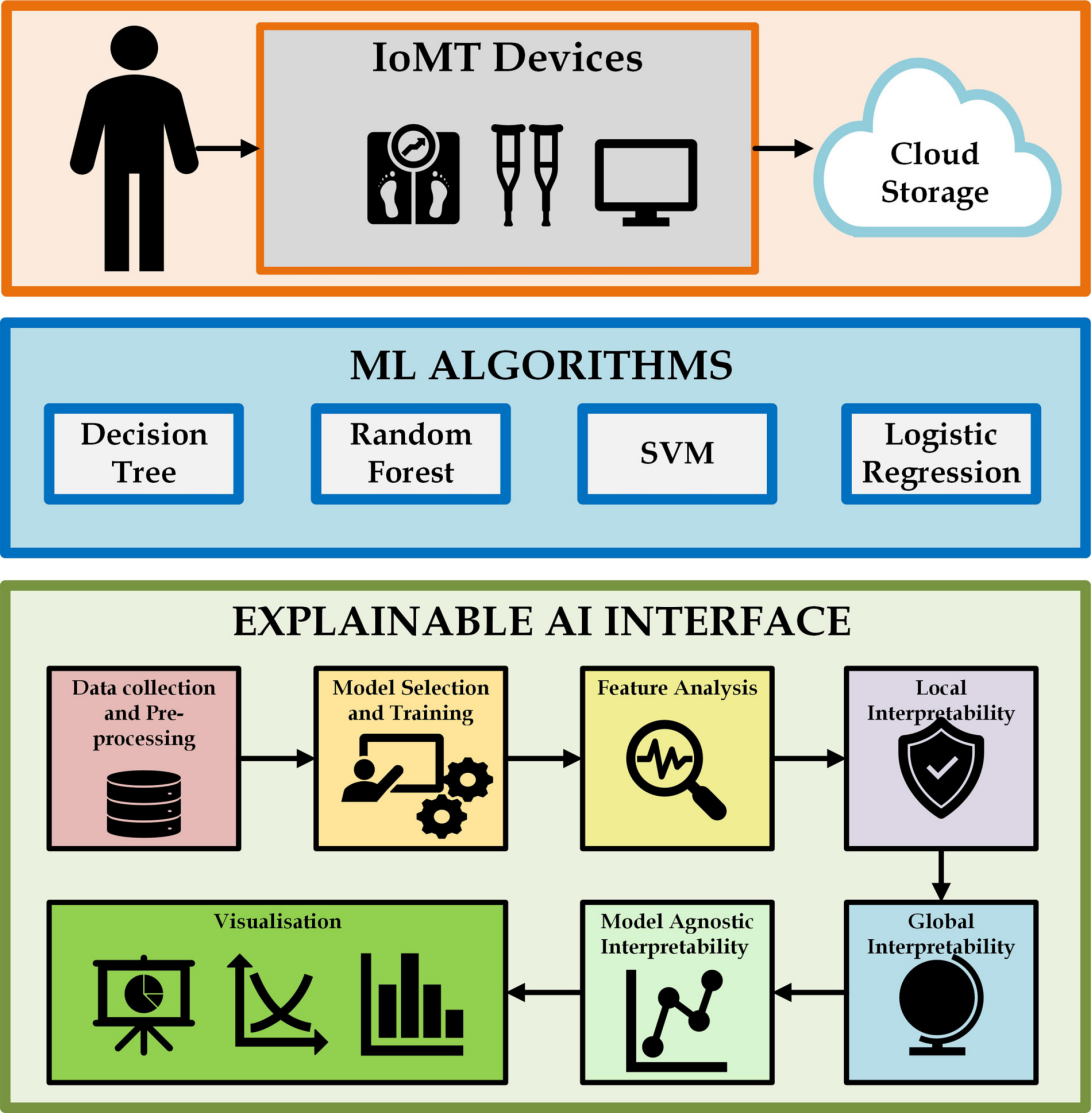
Interfacing ML algorithms with XAI. Creating using Microsoft Visio.

Figure 6 provides an overview of interfacing the ML algorithms discussed in this section with XAI. In terms of monitoring and managing cardiovascular health, IoMT devices play an important role in the use of advanced transformation techniques. These devices increase power connectivity, analyze data, and monitor remotely, providing advanced care for cardiac patients and improving patient health. There are many IoMT devices for monitoring the heart behavior of patients, such as remote ECG monitors, wearable heart rate monitors, pacemakers, BP monitors, temperature monitors, and medication dispensers. These devices help healthcare professionals to monitor patients continuously. They can personalize treatment plans, and they can easily predict previous symptoms and take immediate action.

Machine learning techniques play a substantial role in identifying heart diseases with the help of IoMT devices. Data are collected from IoMT sensor devices, and ML algorithms understand the data, detect anomalies, and produce solutions for accurate heart diagnosis. Many ML algorithms can be applied to train the model to analyze data and recognize patterns. A large volume of data can be processed by ML algorithms from IoMT sensor devices, such as blood pressure measurements, ECG reading results, and heart rate information. ML algorithms like decision trees, Random Forest, SVM, and Logistic Regression are applied here with IoMT devices.

AI algorithms suggest transparent and interpretable explanations for making decisions or predictions. Traditional AI algorithms work as black boxes and produce results with less transparency. When we use explainable AI, it produces an understanding of the reasoning behind its results. Data are collected from IoMT devices and sent for preprocessing, followed by model selection and training. After training the data, feature analysis is done, and during local interpretability, explainable AI uses LIME to understand how specific features contribute to identifying the heart disease. As global interpretability, SHAPLEY helps explainable AI analyze overall behavior and features and their relationship to the decision-making process. Model-agnostic interpretability independently understands the prediction process and aims to apply it to any algorithm. The results can finally be visualized so that appropriate decisions can be made.

### 3.4 Mathematical modeling

#### 3.4.1 Random Forest

Random Forest (127) is an ensemble technique of a machine learning algorithm applied for classification and regression problems. The ensemble combines many models to make predictions accurately. To make an accurate prediction, a Random Forest combines many decision trees (128). Forest refers to a collection of decision trees. Every tree is made independently by a subset of the training data and its input features. Selecting data and features randomly reduces the overfitting problem and creates diversity among each tree. Random forest considers the majority vote from different samples of the decision trees for classification and regression tasks. Bagging or bootstrap and boosting are the two types of ensemble methods. Bagging depends on majority voting by creating many training subsets from the training sample with replacements. Boosting refers to joining weak and strong data by making sequential models to produce the highest accuracy. When the amount of data in the training set is n, then with replacement “n,” data are sampled at random as a bootstrap sample. This helps to grow the tree with training data. When there are “m” input variables, v<m is chosen so that “v” variables are taken at random from “m.” The value “v” is constant when the tree grows to the maximum extent. Many subtrees made by the parameters are formed in the forest. When the forest is completely trained for classification, it is traversed across all the subtrees (129). The classification result from each tree is taken as a vote. The maximum vote is considered a new instance. The generalization error ($PE^{*}$) for the Random Forest is given by Equation 1.

(1) $${PE}^{*}=\;P_{x,y}\left(mf\left(X,Y\right)\right)<0$$

Here, mf(X,Y) is a margin function that measures the average number of votes from (X,Y) exceeding any other class. X refers to the prediction variable and Y refers to the classification task. “I” denotes the indicator function. The expected value for the margin function of a random forest is indicated as Equation 2.

(2) $$R=\;E_{X,Y}\left(mf\left(X,Y\right)\right)$$

A Random Forest’s average strength and the base classifiers’ mean correlation are joined as generalization errors. If ρ represents the mean rate of correlation, the generalization error value for the upper bound is given by Equation 3.

(3) $${PE}^{*}\leq \rho (1-s^{2})/s^{2}$$

To achieve better accuracy in a Random Forest, the subtrees of decision trees must be consistent and diverse. Random Forest is very efficient in detecting outliers. It is scalable, robust, and handles missing data without imputation.

#### 3.4.2 Local Interpretable Model-Agnostic Explanations

LIME (130) is a *post-hoc* model-agnostic framework for any black box machine learning model’s judgment for all instances (55). LIME creates new data from the nearest neighborhood and finds the predictions of these new samples with the help of a black box model. LIME’s explanation depends on monitoring the classifier model’s behavior based on local surrogate models. The LIME algorithm follows three steps to train a surrogate model.

1. Select a few data instances as $x\in R^{d}$, representing the reason for an opaque recommender model f predicting the feature vector x for the probability f(x). LIME expects the data to be converted into an interpretable picture like a binary vector $x^{\prime }\in \{0,1{\}}^{d^{\prime }}$ representing the available/non-available components.
2. Create a new dataset Z of perturbed data $z^{\prime }\in \{0,1{\}}^{d^{\prime }}$ by taking non-zero elements of $x^{\prime }$ at random. The labels must be identified for this new set of data elements in Z in the closest area of $x^{\prime }$. To obtain the labels for the new data, the perturbed samples $z^{\prime }\in \{0,1{\}}^{d^{\prime }}$ are transformed back into the original form $z\in R^{d}$. The opaque model f is then examined for each instance f(z). Because the perturbed samples $z^{\prime }$ are randomly generated, there might be z samples that are closer or farther away from the original instance x for weighing. This weight is measured as $\Pi_{x}(z)$ to evaluate the closeness between the data x and z.
3. Using this newly weighted data Z and the labels created by f(z), a new model g∈G is trained, where G refers to models such as decision trees, linear models, and so on. The interpretable and explanatory surrogate model ξ(x) of the new data g is then used to explain f(x) as shown in Equation 4.
  (4) $$\xi \left(x\right)=g\in G\;\arg\min\left(L(f,g,\Pi_{x})+\Omega (g)\right)$$
  Here, L is the loss function, which measures how g follows the behavior of f in the nearest neighborhood of x. Minimizing this loss function ensures that the behavior of g aligns with the behavior of f indicated by $\Pi_{x}$. The complexity of the model Ω(g) must be kept low. When $g(z^{\prime })$ is represented as a linear function, $g(z^{\prime })=\varphi^{T}z^{\prime }+\varphi_{0}$, the equation 5 becomes a linear regression problem to evaluate φ and $\varphi_{0}$.
  (5) $$L(f,\varphi_{0},\Pi_{x})=\sum_{z,z^{\prime }\in Z}\Pi_{x}(z){\left(\;f\;(z)-(\varphi_{0}+\varphi^{T}z^{\prime })\right)}^{2}$$
  The advantages of LIME are that it is easy to implement, completely fast in terms of computational techniques, and easy to work with in tabular data, text, and images.

#### 3.4.3 SHAPELY Additive exPlanations

The SHAP (124) method improves computational time, and tree-based methods improve explanation precision. The main goal of SHAP is to form perturbations to simulate the features that are not present and to use the linear local model to approximate the prediction changes as given in LIME. It ignores retraining the model without the feature of interest. Local explanations can be combined to describe the model’s global performance. Local and global explanations are reliable with each other as they follow the same basic methods. SHAP uses agnostic explainer KernelSHAP and model-specific explainers such as TreeSHAP for tree-based models, DeepSHAP for deep models, and LinearSHAP for linear models.

SHAP produces SHAPELY values, which express model predictions as linear combinations of binary variables. This framework explains how each covariate contributes when fixed in the model. The prediction f(x), using $s(x^{\prime })$, for a linear model for the binary values $y^{\prime }\in \{0,1{\}}^{M}$ with the elements $\emptyset_{i}\in R$, is given by Equation 6.

(6) $$s{(y}^{\prime })=\;\emptyset_{0}+\sum_{i=1}^{M}\emptyset_{i}y_{i}^{\prime }$$

Here, M is a variable for explanations which is shown in Equation 7.

(7) $$\Phi_{i}(f,z)=\sum_{y^{\prime }\subseteq z^{\prime }}\frac{\left(|y^{\prime }|!(M-|y^{\prime }|-1)!\right)}{M!}[f_{x}(y^{\prime })-f_{x}(y_{i}^{\prime })]$$

where f is the model of this method, z is the variable, and $z^{\prime }$ are the selected variables. The value $f_{x}(y^{\prime })-f_{x}(y_{i}^{\prime }$ denotes, for every prediction, the SHAPELY values from its mean value of the ith variable. Local accuracy results from the explainable model are equal to those of the basic models. The missing nature of the SHAPELY values has features that were not added as the first input without any effect. Consistency of the model changes with reliance on a single feature, and related characteristics cannot be reduced independently of other factors. The advantage of SHAP is that it predicts an instance disseminated among the feature values. The limitations are its slow computational time, high computational complexity, and problems with explanation instability similar to LIME.

### 3.5 Algorithm

This section describes two algorithms, one for the heart risk evaluation through Algorithm 1 and the other for explaining heart failure through the Algorithm 2. These two algorithms comprehensively analyze and explain the risk of Heart Failure as a complete solution. In Algorithm 1, the performance metrics such as accuracy, precision, recall, sensitivity, specificity, and F1-score are evaluated. During the testing phase, when $x_{t}est$ becomes 1, the heart failure alarm will be activated. Otherwise, the result indicates that the function of the heart is normal. In Algorithm 2, the model with local surrogates explains the appropriate decision after heart failure when the prediction is local. In case the probability of the prediction is global, explainability is achieved in global surrogates.

**Algorithm 1:** Algorithm for heart disease prediction.

**Input**: $x=[\sum_{i=0}^{n}I_{n}$; $x_{t}rform=label.encoder(x)$; $y\leftarrow y_{train},y_{test}$; $x\leftarrow x_{train},x_{test}$; $n\leftarrow samples_{o}f_{i}mages$; $TRUE_{P}\leftarrow TruePositive$; $TRUE_{N}\leftarrow TrueNegative$; $FALSE_{P}\leftarrow FalsePositive$; $FALSE_{N}\leftarrow FalseNegative$; Features←a,b; **Accuracy**: $\frac{TRUE_{P}+TRUE_{N}}{TRUE_{P}+TRUE_{N}+FALSE_{P}+FALSE_{N}}$; **Precision**: $\frac{TRUE_{P}}{TRUE_{P}+FALSE_{P}}$; **Recall**: $\frac{TRUE_{P}}{TRUE_{P}+FALSE_{N}}$; **F1-score**: $\frac{2*TRUE_{P}}{2*TRUE_{P}+FALSE_{P}+FALSE_{N}}$; **Activation**: max[Accu,Prec,Reca,F1−Sco,Sensi,Speci]; **while** yis≠0 **do** **if** $x_{test}$ is Potable **do** $accu\leftarrow \frac{TRUE_{P}+TRUE_{N}}{TRUE_{P}+TRUE_{N}+FALSE_{P}+FALSE_{N}}$; $Preci\leftarrow \frac{TRUE_{P}}{TRUE_{P}+FALSE_{P}}$; $reca\leftarrow \frac{TRUE_{P}}{TRUE_{P}+FALSE_{N}}$; $f1\text{-}sco\leftarrow \frac{2*TRUE_{P}}{2*TRUE_{P}+FALSE_{P}+FALSE_{N}}$; $Sensi\leftarrow \frac{TRUE_{P}}{TRUE_{P}+FALSE_{N}}$; $Speci\leftarrow \frac{FALSE_{P}}{FALSE_{P}+TRUE_{N}}$; **end** **else** $x_{test}$ is Not Potable $accu\leftarrow \frac{TRUE_{P}+TRUE_{N}}{TRUE_{P}+TRUE_{N}+FALSE_{P}+FALSE_{N}}$; $Preci\leftarrow \frac{TRUE_{P}}{TRUE_{P}+FALSE_{P}}$; $reca\leftarrow \frac{TRUE_{P}}{TRUE_{P}+FALSE_{N}}$; $f1\text{-}sco\leftarrow \frac{2*TRUE_{P}}{2*TRUE_{P}+FALSE_{P}+FALSE_{N}}$; $Sensi\leftarrow \frac{TRUE_{P}}{TRUE_{P}+FALSE_{N}}$; $Speci\leftarrow \frac{FALSE_{P}}{FALSE_{P}+TRUE_{N}}$;

**Algorithm 2:** Algorithm for explainable AI.

**Input**: $x=[\sum_{i=1}^{c_{n}}x_{n}(1-x_{n})]$; $y\leftarrow y_{train},y_{test}$; $x\leftarrow x_{train},x_{test}$; k←No_of_samples; cf←Complexityfunction; r←local_surrogate_regressor; L←Loss_function; d←Permutations; d∪D; E←Number_of_players; v←value_function_of_the_players; **while** Y≠Local **do** **if** Predictproba is local **then** L(x)←Loss_function; $\exp\leftarrow \theta ()k=L(r,cf,\pi_{k})+\omega (cf)$; ▹**Decision Explained with Local Surrogates (LIME)** **else** Predictproba is global θ(y)←Costfunction; $exp\leftarrow \theta (v)=\sum_{d\subset Ei}|d|!(E-|d|-1)!/E!*(v(E\cup i-V(p)))$; ▹**Decision Explained with Global Surrogates (SHAPELY)** **end**

### 3.6 Environment-based attribute access control algorithm

The dataset under consideration must be protected and authenticated. Hence, rigorous data access control permissions must be set in the cloud to access it properly. A secure environment-based attribute access control system is required in this context to protect unauthorized access to the data in the cloud. The model is divided into two categories: static and dynamic. Users with the lowest role, such as those looking for recommendations, access information in a static environment. This audience will only be permitted to obtain legal information; no other transactions will be permitted. In a dynamic state, different parameters are measured and recorded at various instances of time. Thus, many data acquisition and update cycles are a series of transactions carried out in the cloud in big time. These states only allow special users such as clinicians and administrators.

The development of a digital identity is the first step. The key used in the digital identity protects and guarantees a transmission between the server and the client, and the key is $p_{k}$. The user shares its digital account identity and the symmetric key $e_{k}$, and the corresponding data information can be obtained by decrypting the key $p_{k}$. Various functions used in the algorithm, such as IssueRole, revokeIssueRole, and partialExtension, help the framework achieve a secured space to function. After the digital identity is authenticated and a role is identified, the model can access the framework accordingly. Each of the entity’s transactions is considered along with its authorization. Therefore, a secure environment for the fuzzy framework is achieved.

## 4 Results

### 4.1 Experimental setup

The 11 parameters that determine the failure of the heart are acquired from various sources across various countries and used in this work. These parameters have a strong influence on determining heart failure in real time. Most of these parameters are embedded with IoMT sensors, which can be integrated through information fusion in cloud platforms. Later, these data are classified by cloud machine learning models and transformed into a valid dataset. One such dataset is used in this work for experimental analysis. Because the problem is binary, the experimentation is done with machine learning models such as SVM, Logistic Regression, Decision Tree, and Random Forest. The explanation of this dataset is provided by LIME and SHAPELY values. The classification probability of the random forest model is evaluated due to its high classification accuracy with various explanations for clarity.

### 4.2 Results

#### 4.2.1 Preprocessing

**Figure 7:**
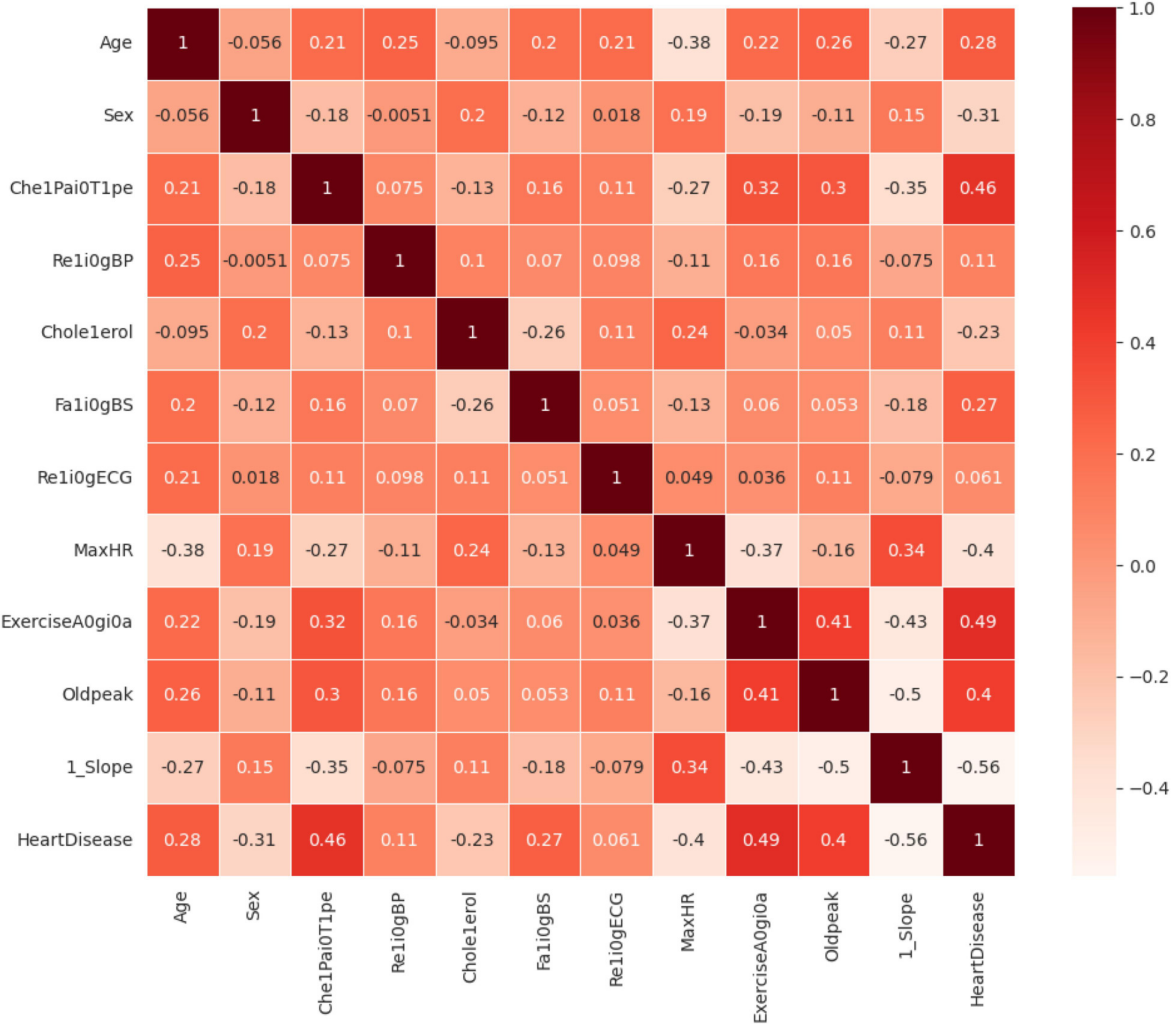
Correlation matrix of various features.

The dataset is preprocessed to convert the data types into a unified format, which makes it suitable for the classification problem. The statistical analysis of the various features of interest is tested with the correlation matrix shown in Figure 7. The features that have a higher correlation as per the correlation map are Exercise-Induced Angina, Chest Pain Type, and Age. **Preprocessing equations**

1. Missing value imputation
  Let $X=\{x_{1},x_{2},\ldots ,x_{n}\}$ be a feature vector with missing entries.
  (11) $$x_{i}=\left\{ \begin{array}{cc} x_{i}\; & \text{if}\;x_{i}\;\text{is not missing}\;\\ \bar{x}\; & \text{if}\;x_{i}\;\text{is missing}\; \end{array}\right.$$
  where $\bar{x}=\frac{1}{n^{\prime }}\sum_{\;j=1}^{n^{\prime }}x_{j}$ is the mean of observed values and $n^{\prime }$ is the number of non-missing entries. Mean imputation replaces missing values with the average of the available values in the feature.
2. Label encoding
  Let a categorical variable $C\in \{c_{1},c_{2},\ldots ,c_{k}\}$ be transformed into integer labels as Equation 12.
  (12) $$\text{Label}(c_{i})=l_{i}\;\text{, where}\;l_{i}\in \{0,1,\ldots ,k-1\}$$
  Each distinct category $c_{i}$ is mapped to a unique integer $l_{i}$. This method is commonly used when categories have no intrinsic ordering.
3. Standardization of binary target class
  Given a binary target variable y∈{0,1}, standardization is defined as Equation 13.
  (13) $$y_{\text{std}}=\frac{y-\mu_{y}}{\sigma_{y}}$$
  where
  $$\mu_{y}=E[y]=p,\;\sigma_{y}=\sqrt{\;p(1-p)}$$
  Assuming y∼Bernoulli(p), the mean $\mu_{y}$ and standard deviation $\sigma_{y}$ are computed to transform y into a zero-mean, unit-variance variable suitable for certain learning models.

#### 4.2.2 Machine learning models

The target attribute Heart Disease is a binary classifier where “1” indicates heart failure and “0” indicates no failure. Because the problem is binary, we apply machine learning models such as SVM, Logistic Regression, Decision Tree, Random Forest, AdaBoost, and Gradient Boosting Classifier Algorithm. Model parameters and specifications of various methods are specified in Table 5. The model parameters of the Random Forest are slightly higher than that of the other models with respect to AUC. The results obtained in this work have only a thin difference in the metric values measured across various machine learning models since the dataset is free from missing values or class imbalance. The cost function of Logistic Regression (Equation 8), Gradient Boost (Equation 9) AdaBoost (Equation 10) are highlighted. The metric evaluation is presented in Table 6. There are essential metrics such as sensitivity and specificity, which estimate the true positive rate $T_{PR}$, true negative rate $T_{NR}$, false positive rate $F_{PR}$, and false negative rate $F_{NR}$. These parameters calculate the reliability of the model. The explanation of a machine learning model is based on reliability and performance. Table 7 presents these metrics with corresponding values for each machine learning model.

**Table 5:** Model parameters and specifications.

Model	Hyperparameters	Time complexity	Cost function
Logistic Regression (131)	Solver, penalty (optional)	2–3 s	$\;\frac{e^{\alpha +\beta x}}{1+e^{\alpha +\beta x}}\;(8)$
SVM (132)	C Gamma Kernel size	2–3 s	$\left[W(\alpha )=\sum_{i}\alpha_{i}-\frac{1}{2}\sum_{i,j}y_{i}y_{j}\alpha_{i}\alpha_{j}\phi (x_{i})\cdot \phi (x_{j})\right]$
Decision Tree (133)	Gini, max depth, minSamples, features	2–3 s	• Find best split $s^{*}$ in all variables that maximize impurity decrease • Label the currentNode with the best-split variable and its value • Divide the available learning data L into $L_{l}$ and $L_{r}$ • Create nodes $t_{l}$ and $t_{r}$ that contain data $L_{l}$ and $L_{r}$, respectively • Repeat with $currentNode=t_{l}$ and data $L_{l}$ • Repeat with $currentNode=t_{r}$ and data $L_{r}$
Random Forest (127)	max_depth Min_sample_split Max_leaf_nodes Min_samples_leaf N_estimators Max_sample (bootstrap sample) Max_features	2–3 s	• There are M number of trees instead of only one tree • There are p number of variables in each tree instead of k, where p≤k and k is the total number of variables • Each tree is built using $\tilde{N}$ number of samples, where $\tilde{N}$ is 63.2% of the total number of samples N
Gradient Boost (134)	Maximum iterations Learning rate Maximum depth Or maximum leaf nodes	2–3 s	$\;\text{MSE}=\frac{1}{N}\sum_{i=1}^{N}(y_{i}-{\hat{y}}_{i}{)}^{2}\;(9)$
AdaBoost (135)	Number of estimations Learning rate	2–3 s	$\;\text{Exponential Loss}=\sum_{i=1}^{N}\exp(-y_{i}\cdot {\hat{y}}_{i})\;(10)$

**Table 6:** Classification report of the various machine learning models.

Method	Accuracy	Precision	Recall	F1-score	MCC	ROC
SVM (132)	0.89	0.89	0.89	0.89	0.777	0.94
Logistic Regression (131)	0.875	0.875	0.875	0.874	0.746	0.933
Decision Tree (133)	0.961	0.962	0.961	0.961	0.922	0.991
Random Forest (127)	0.955	0.955	0.955	0.955	0.910	0.994
Gradient Boost (134)	0.935	0.935	0.935	0.935	0.868	0.985

**Table 7:** Sensitivity and specificity analysis of the various machine learning models.

Method	Sensitivity	Specificity
SVM (132)	0.89	0.89
Logistic Regression (131)	0.875	0.875
Decision Tree (133)	0.962	0.961
Random Forest (127)	0.955	0.955
Gradient Boost (134)	0.935	0.935

#### 4.2.3 Tenfold classification

Table 8 illustrates the results from 10-fold validation without preprocessing using a Python IDE. Without the application of preprocessing, the results provide accuracy, which is comparatively less than the original 70-30 train-test evaluation. The model has already been optimized with the highest levels of accuracy through preprocessing techniques. The preprocessed values are already tabulated in Table 6.

**Table 8:** Classification report of the various machine learning models for 10-fold.

Method	Accuracy	Precision	Recall	F1-score	AUC
SVM (132)	0.844	0.844	0.844	0.844	0.904
Logistic Regression (131)	0.861	0.860	0.860	0.861	0.924
Decision Tree (133)	0.792	0.792	0.794	0.792	0.778
Random Forest (127)	0.859	0.859	0.859	0.859	0.920
AdaBoost (135)	0.781	0.781	0.782	0.781	0.780
Gradient Boost (134)	0.874	0.873	0.873	0.874	0.928

#### 4.2.4 Explainable AI models

The Random Forest model is selected to explain the LIME and SHAPELY models of the XAI. The LIME model explains the local surrogates and estimates which features are positive (increase) and which are negative toward the prediction of the target class. This model is used in a local surrogate for a particular dataset instance. This application also determines the feature weights and prediction score for each classifier in accordance with a specific instance.

SHAPELY uses various models based on the explainer suggested by Random Forest. It provides the testpatch, which distributes features in the global surrogates. Then, SHAPELY uses plots like summary plot, which provides the order of the features that determine the magnitude of the output. It also provides the dependency plot, which explains the dependency between the two variables of interest in global surrogacy. The decision plot of SHAPELY provides the decision on a particular instance and explains the rationale behind the classification with the feature impact analysis.

**Figure 8:**
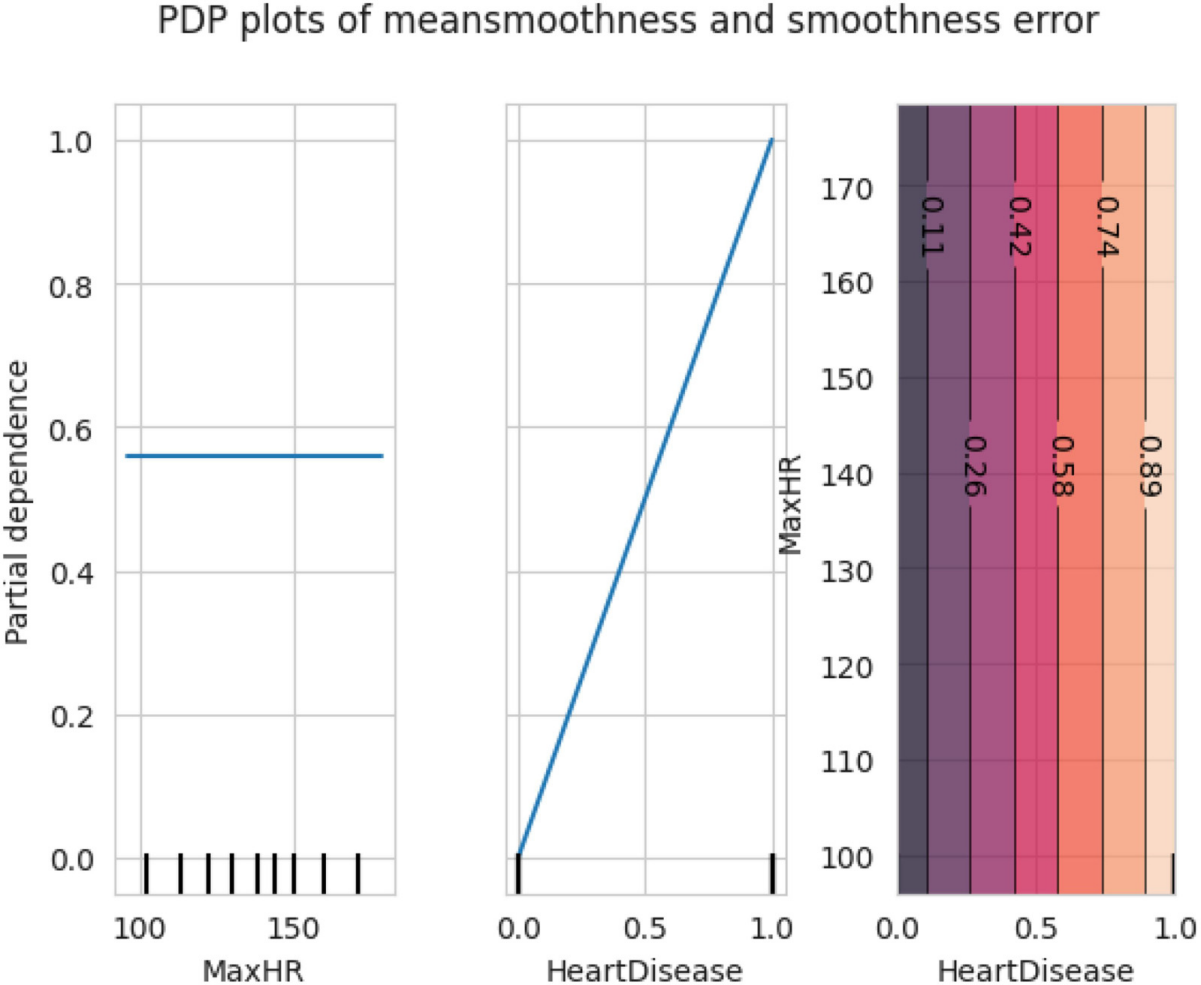
Partial dependency plot between the MaxHR and heart disease.

**Figure 9:**
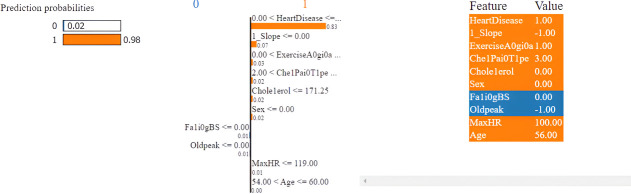
LIME explainer explanation for heart disease prediction with NoteBook.

**Figure 10:**
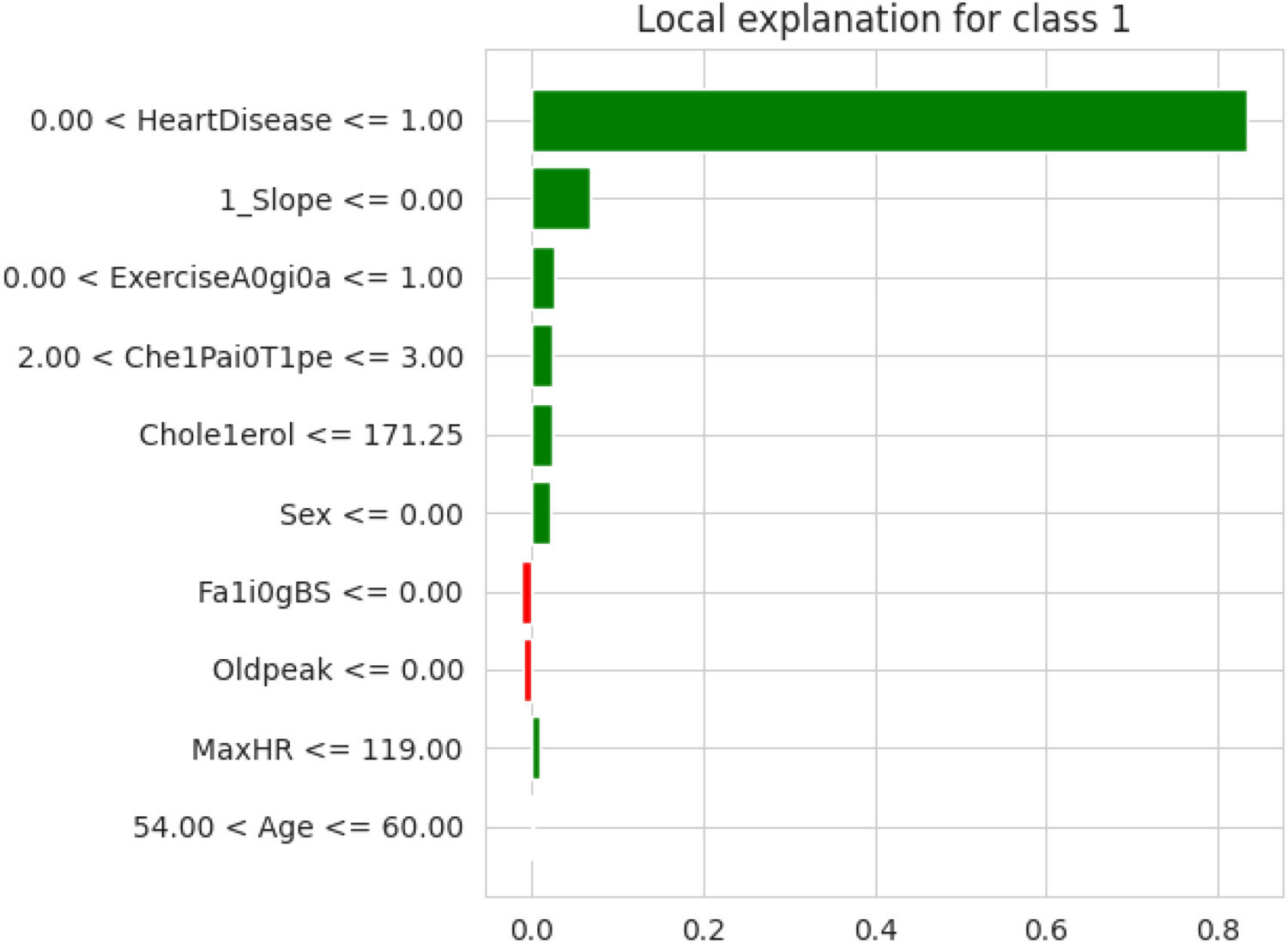
LIME explainer explanation using PyPlot for feature significance.

The first model discussed for explainability is the partial dependency plot (PDP). This plot shows the relationship between the two contributing features through linear relationship estimation through LASSO. The correlation between the two attributes is represented by the PDP. The plot between the MaxHR with the target feature Heart Disease is presented by the PDP plot in Figure 8. The LIME model predicts the behavior of an instance in the local surrogacy and explains the relationship between the target attribute and the rest of the features in the dataset. This also estimates the attribute weights, which features provide a positive relationship to the target prediction, and which features provide a negative response. According to an instance depicted in Figure 9, class 0, which is no disease, has a 2% probability, and class 1, which is the Heart Disease, has a 98% probability of occurrence. This notebook model explains the list of the features that influence the target attribute. Figure 10 shows the Pyplot, which describes the features that have a positive relationship towards the target, such as 1_slope, Chest Pain Type, Age, Cholesterol, Blood Sugar, Exercise Angina, Sex, and MaxHR. The features with a negative relationship to the target, like Old Peak and Blood Sugar, are also explained. Using linear relationships, LIME thus explains the relationship between the target attribute and the rest of the attributes in a particular row instance. This also estimates the feature weight, nature, and significance of that particular local surrogacy. The SHAPELY explainer provides local and global surrogate explanations for the local instance and the complete dataset, respectively. It uses various plots to describe each feature’s significance in determining the target’s magnitude. The plots that are depicted in this work include

• Force plot
• Test patch
• Dependency plot
• Summary plot
• Decision plot

**Figure 11:**
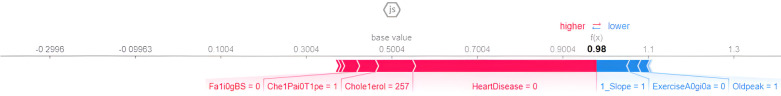
SHAPELY explainer explanation for heart disease with a force plot.

**Figure 12:**
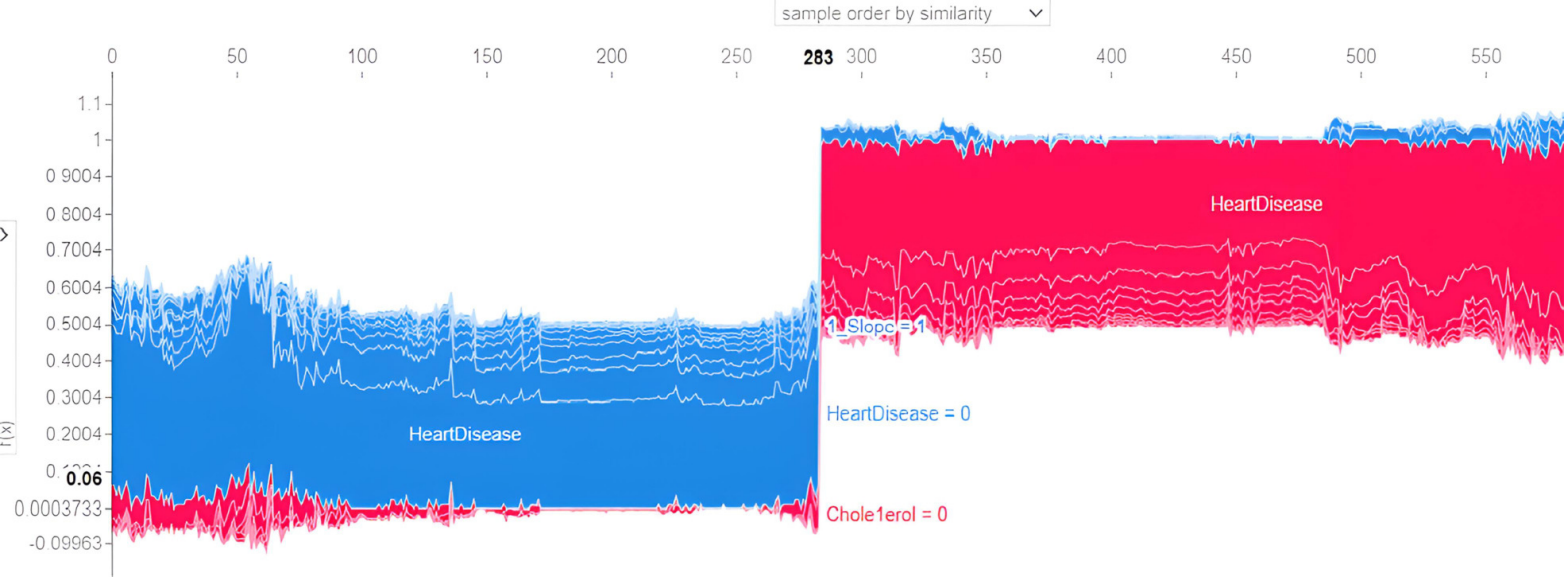
SHAPELY explainer explanation for heart disease with a classic test patch.

**Figure 13:**
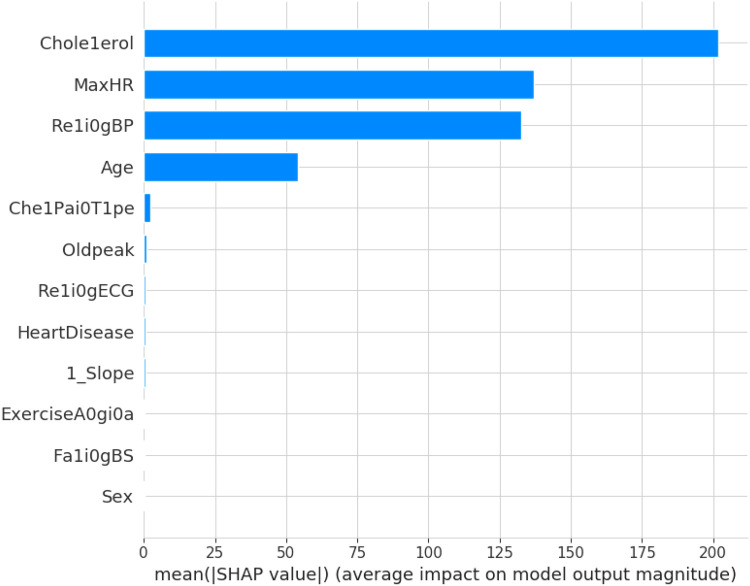
SHAPELY explanation for heart disease with a summary plot.

**Figure 14:**
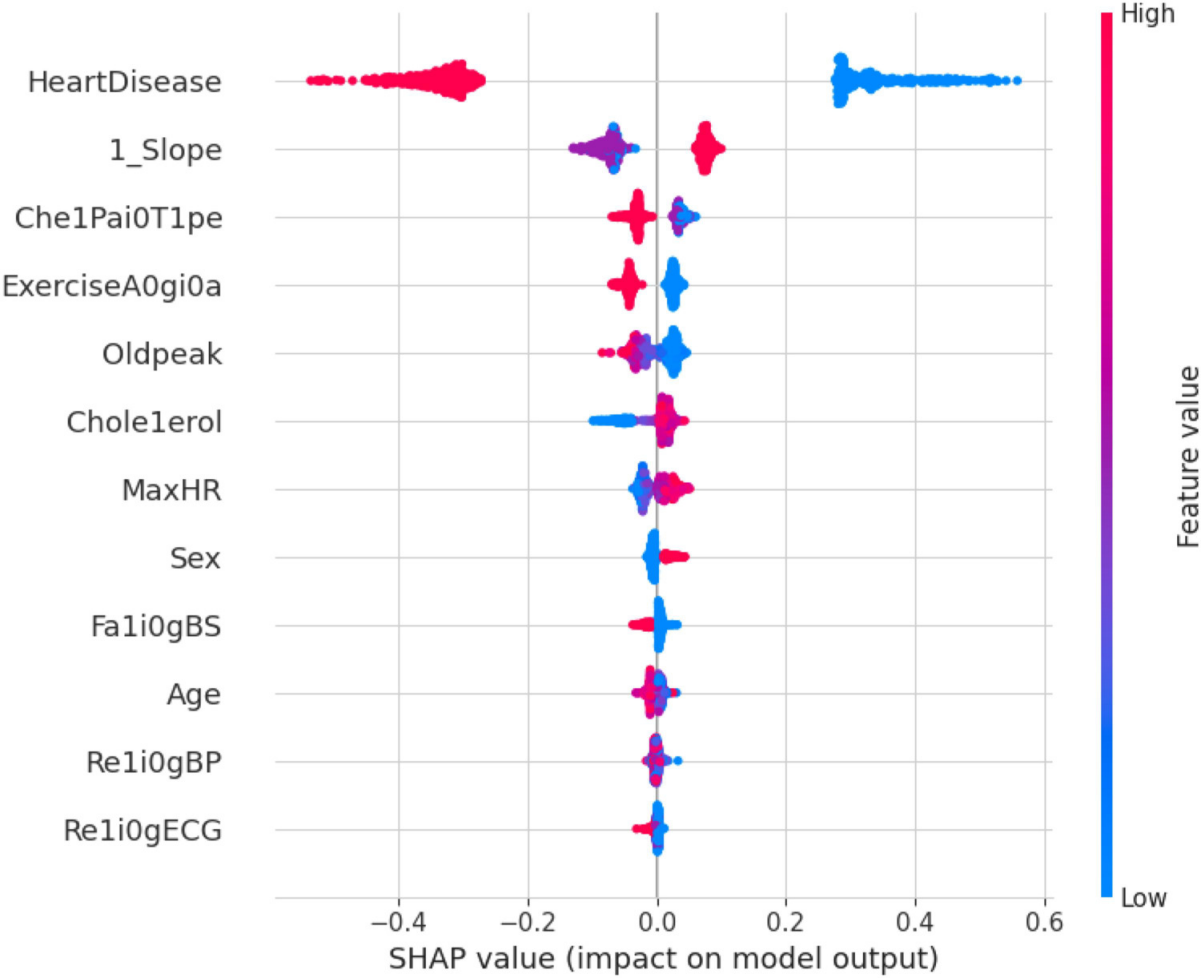
SHAPELY explanation for heart disease with a summary plot.

**Figure 15:**
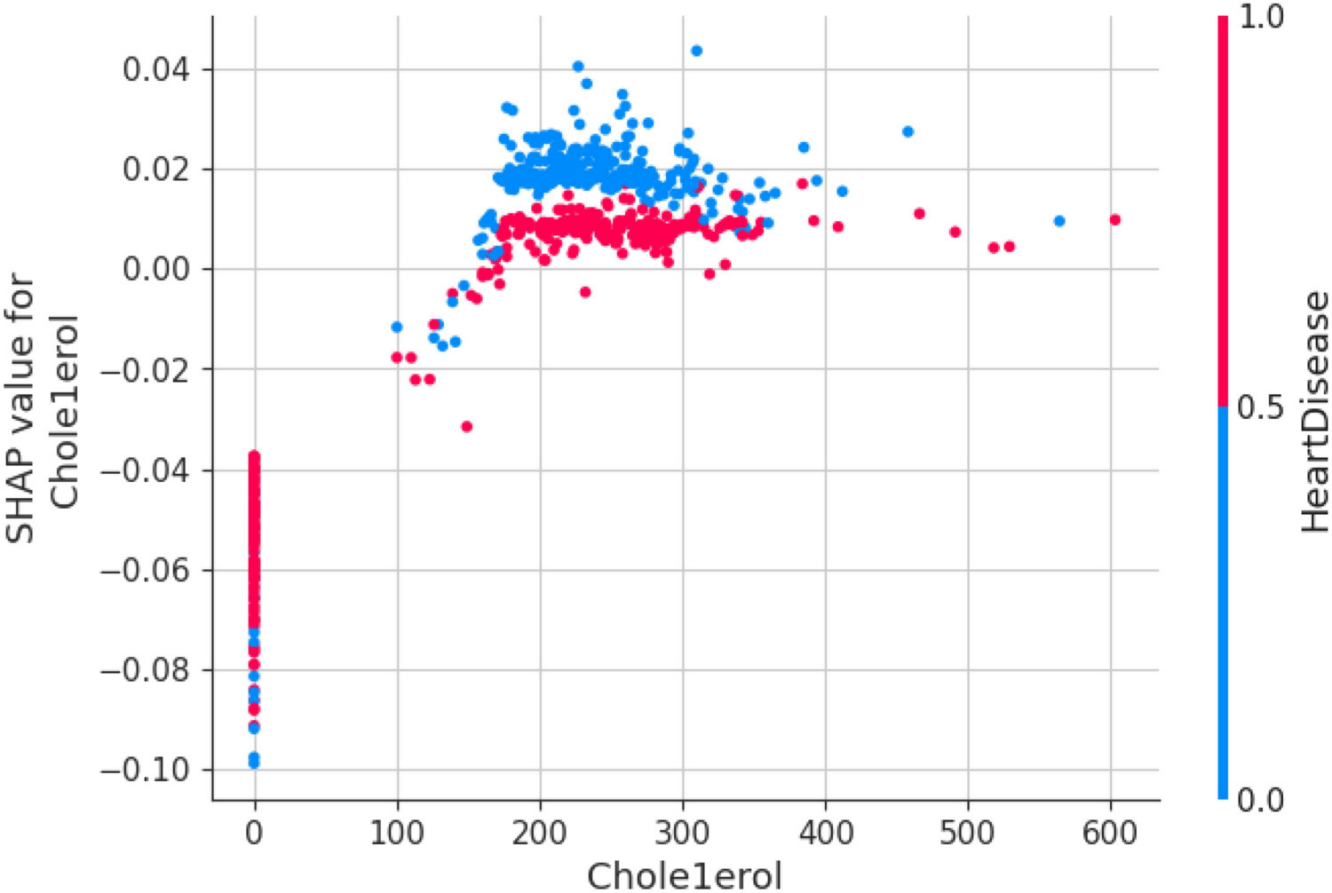
SHAPELY explanation for heart disease with a decision plot.

The force plot explains an instance in the local surrogacy and tells how the feature values take a range between minimum and maximum, with the perception of a corresponding instance. It shows how the features contribute to the model prediction for a specific observation, as shown in Figure 11. The prediction score for this model is 0.98. The red-colored features increase the prediction score, and the blue-colored features decrease the prediction score. The features closer to this dividing region have the highest impact on the model prediction for that particular instance. In this instance, the parameter Cholesterol is for increasing the prediction score and 1_slope for decreasing the prediction. The classic test patch provides the overall distribution of features and shows how they can help predict the target. This global surrogate model explains the entire dataset regarding what features contribute to the prediction of heart failure through a double-colored area. The red color shows chances for Heart Failure, and the blue shows normal output. The classy test patch is described in Figure 12. In this plot, the features closer to the dividing boundary are also highly important in predicting the model. The summary plot lists various features in the dataset and sorts them based on the order of significance in determining the magnitude of the output. Cholesterol, Maximum Heart Rate, Blood Pressure, Age, and Chest Pain Type have the order of significance in determining the target value, respectively. The features and their corresponding weight importance are shown in Figure 13. Figure 14 depicts the summary plot with feature concentration. The target value, Heart Failure, is distributed from 0 to 1. Various features like 1_slope, Chest Pain Type, Exercise Angina, Old Peak, and Cholesterol are plotted as per the order of significance in determining the output magnitude. The red-colored region shows a high impact, and the blue-colored region shows a low impact in predicting the target attribute. The SHAPELY decision plot is illustrated in Figure 15. This is a global surrogate model, where the dependency between the target class and the cholesterol is plotted in the graph in Figure 15. PDP also looks similar to the dependency plot of SHAPELY, but SHAPELY provides granular outputs that can be increased or minimized. The second point is that PDP is only a plot, but a dependency plot is a variable-like result. Taking the average value per variable is like plotting variable importance against the SHAP value, which will look like a PDP graph.

## 5 Discussion

**Figure 16:**
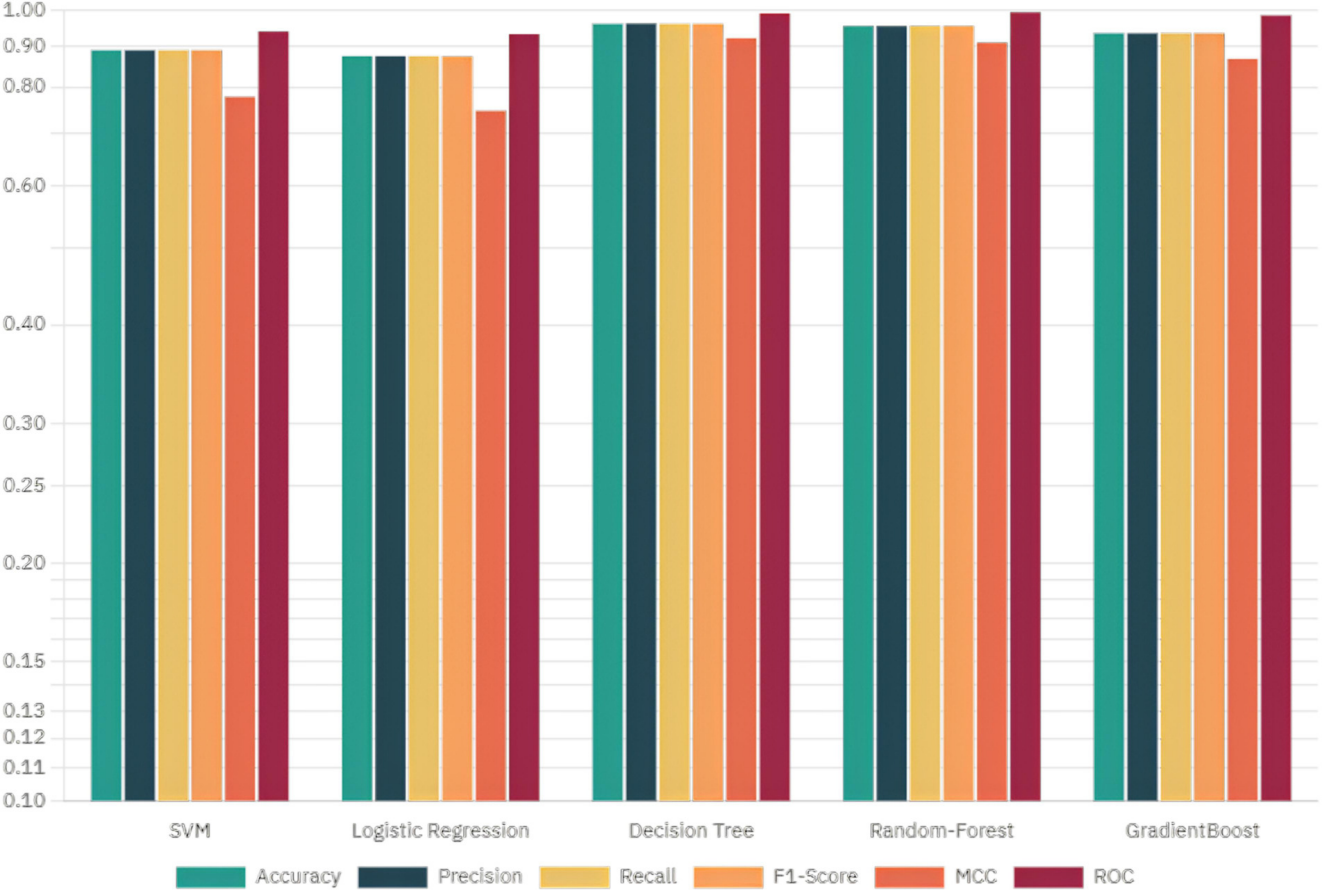
Comparative analysis of the hyperparameters of ML models.

This section deals with the comparative analysis of various machine learning algorithms that are used in this work. This work also deals with how the features contribute to the results in the SHAPELY explainer. The comparative analysis of the various machine learning algorithms is presented in Figure 16. The ratio of rightly predicted data to the total observations determines the accuracy of the model. The ratio of the rightly predicted positive data to the total analyzed positives fixes the precision. The ratio of the rightly predicted positive data to all actual positives is a recall metric. F1-score defines the harmonic mean of both precision and recall. The Random Forest model, which has a higher accuracy of 0.955 and F1-score of 0.955, was selected for explanation by XAI applications. The second-best values for accuracy and F1-score are recorded in the Gradient Boost model with values of 0.935 and 0.935 with a precision of 0.997. Logistic Regression and SVM have accuracy values of 0.875 and 0.875. All these models only have marginal differences in the values of parameters between them. The Decision Tree model recorded a highest accuracy of 0.961, but the AUC was the highest for random forest, which is 0.994. Thus, this model is selected for XAI implementation.

**Figure 17:**
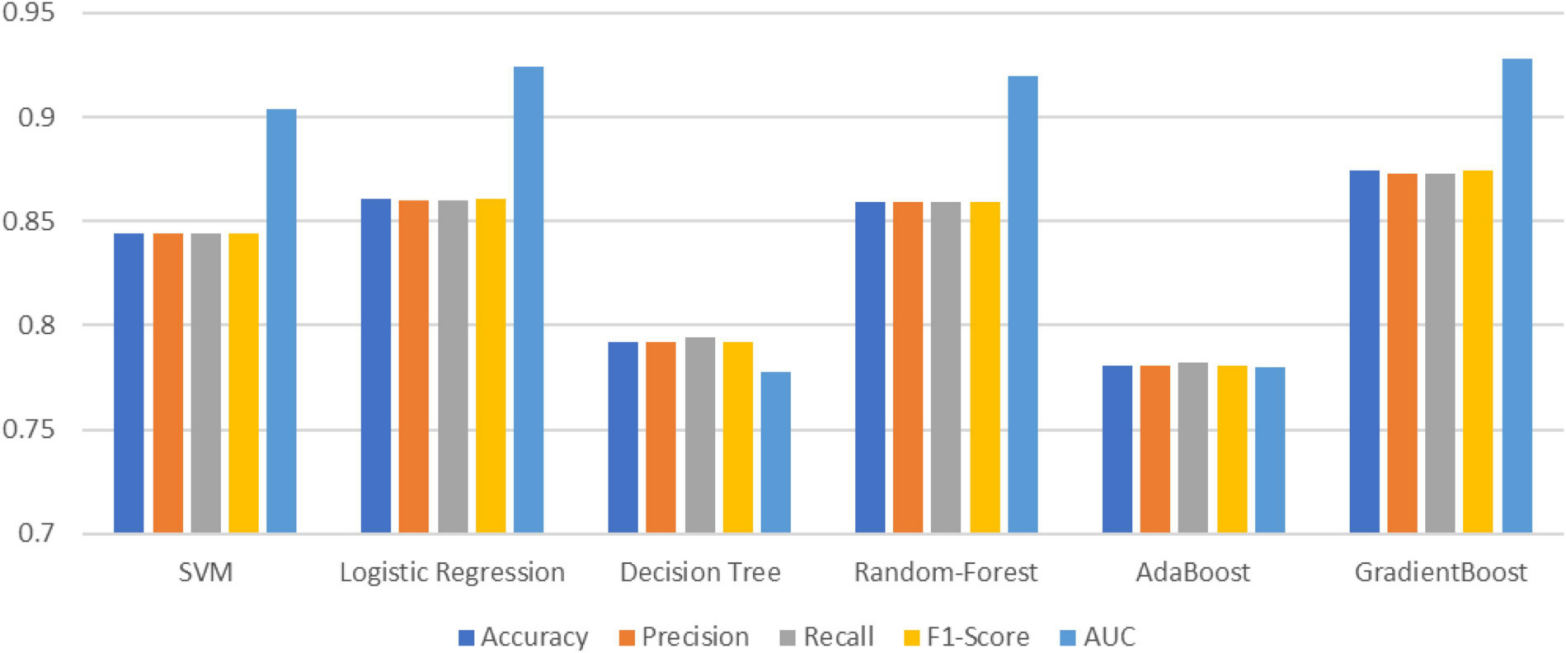
Comparative analysis of the parameters of ML models with 10-fold.

**Figure 18:**
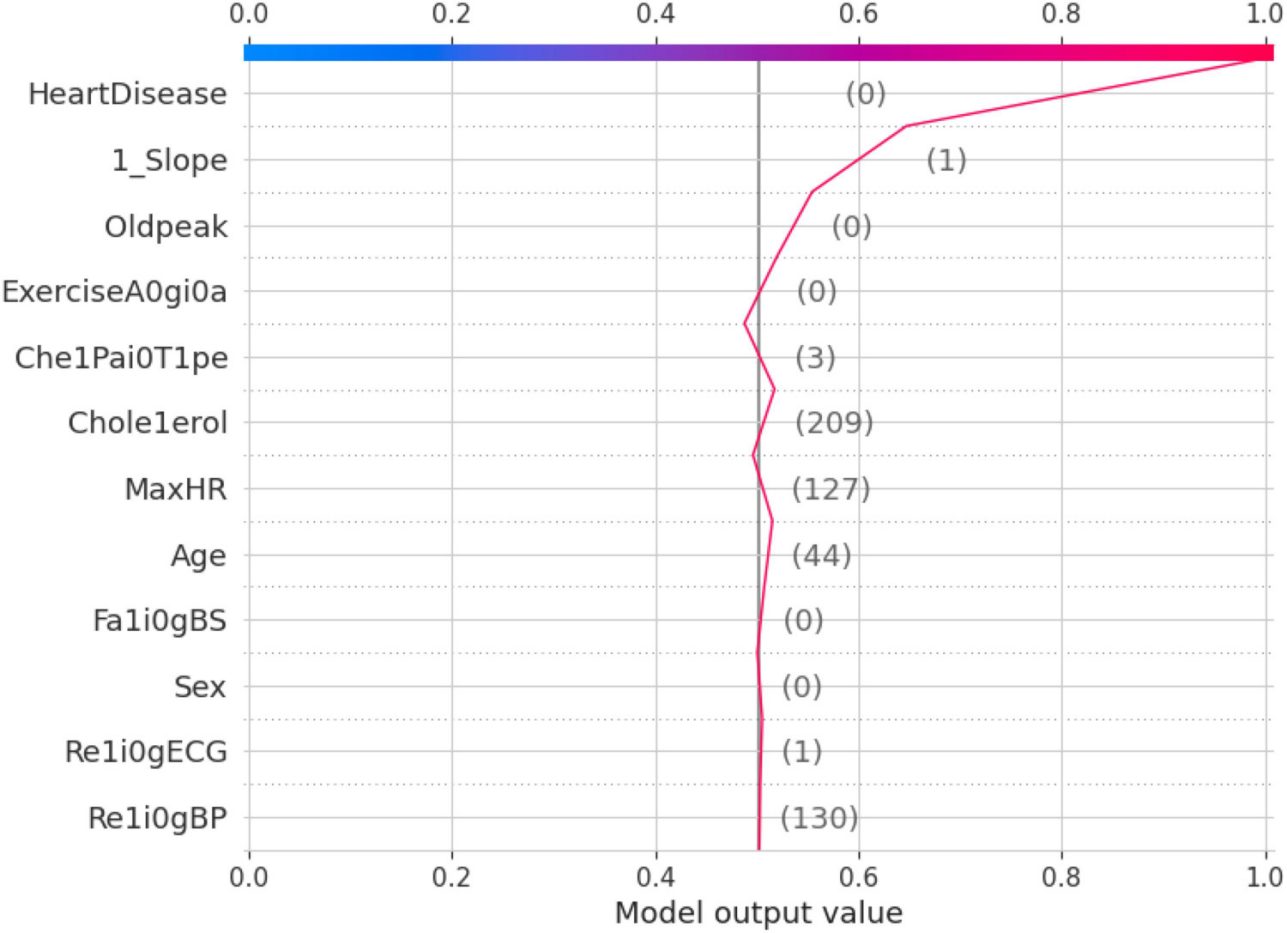
SHAPELY explainer decision plot for heart failure prediction.

**Figure 19:**
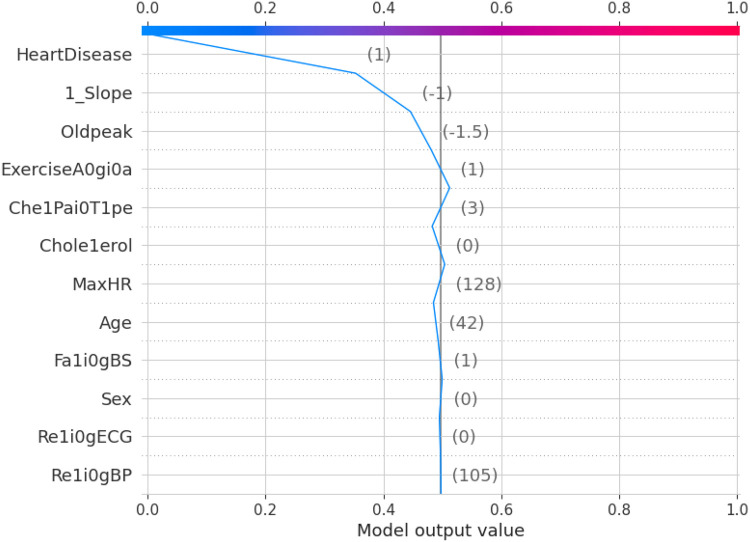
SHAPELY explainer decision plot for normal chest pain.

The 10-fold validation is also presented in Figure 17. These results show reduced accuracy levels with the lack of standard preprocessing techniques. Despite the reduced accuracy levels, Gradient Boosting and Random Forest algorithms perform much better than the rest of the models. The SHAPELY decision plots are presented in Figures 18, 19. These decision plots are extremely important in determining why an instance is classified as normal or abnormal (Heart Failure). In this local instance, the values of 1_slope, ECG Peak, and Exercise Angina are high. The value of cholesterol is also high, and the Chest Pain Type is Recorded as Type 3. All these feature values correspond to the heart disease classification into 1, which means a risk indication of Heart Failure. In the case of Figure 19, all the feature values are normal, and the instance is classified into the normal category. Thus, the decision plot of SHAPELY values provides a detailed explanation regarding how an instance is classified on the basis of various values of the features available.

### 5.1 Challenges

This work has the following challenges (not limited to), which are required to be addressed in the future. The sensors may go out of order and hence can provide false alarms to the cloud and database. The electronic faults may induce false alarms regarding heart failure. The medical data are subjected to be private. Explaining may compromise the privacy and integrity of the individual medical data. Medical data stored in the cloud are vulnerable to attacks if no security mechanisms are provided. If the medical record is stored in a blockchain model, it is extremely difficult to access and explain the same with the XAI model. The reliability of the explanation and privacy need to be enhanced by Federated Learning. Training and demonstration are required for medical practitioners to handle data from wearable sensors and the cloud.

### 5.2 Contributions of the paper

The essential contributions of this paper helps identify the complete purpose of this research. This paper provides a complete illustration of all the sections of IoMT-enabled XAI infrastructure. It also discusses various IoMT applications and case studies related to heart failure in detail. This paper works with a dataset with all the vital parameters required for heart failure prediction. It provides solutions for the explanation of heart failure through local and global surrogates with the explanations of LIME and SHAPELY. This study discusses various state-of-the-art IoMT sensors with practical applicability in medical applications with a discussion of advantages and disadvantages.

### 5.3 Future work

Improvements can be made to this study by applying many advanced techniques. The application of 6G may improve the connectivity and network-related issues associated with wearable sensors. Application of Federated Learning would improve the privacy, reliability, and safety of medical data. Meta-verse applications can enhance IoMT sensor support and provide real-time solutions to heart problems. Industry 5.0 can enhance the quality of service of the proposed system with a human-centric man–machine interface. Web 3.0 standards can provide better semantics, security, and reliability in cloud service.

## 6 Conclusion

Early detection of heart failure is the most desirable and need-of-the-hour application, as the number of cardiac arrest cases increases day by day. A healthy life cycle, clean habits, and a peaceful life are the real medicines to overcome heart disease. Clinical efforts are merely supplementary but not primary in nature in addressing the issues related to heart failure. The IoMT integrated Heart Failure prediction model discussed in this study is extremely useful in this stressful modern-day life. The IoMT sensors can control and monitor most of the parameters relevant to heart failure at the primary level. XAI provides excellent support to this system by indicating what body parameters influence the heart failure condition through various models that show the significance of the features for the prediction of the target. The probability of prediction of the Random Forest model is used by LIME, the local explainer, and SHAPELY, the global explainer, for explaining models related to heart failure prediction. This model is a whistleblower to many such systems developed to make human life longer, better, and safer.

## Data Availability

The original contributions presented in the study are included in the article/Supplementary Material, further inquiries can be directed to the corresponding author.
